# Effects and Optimal Dose of Exercise on Endothelial Function in Patients with Heart Failure: A Systematic Review and Meta-Analysis

**DOI:** 10.1186/s40798-023-00553-z

**Published:** 2023-02-04

**Authors:** Laura Fuertes-Kenneally, Agustín Manresa-Rocamora, Carles Blasco-Peris, Fernando Ribeiro, Noemí Sempere-Ruiz, José Manuel Sarabia, Vicente Climent-Paya

**Affiliations:** 1 Institute for Health and Biomedical Research of Alicante (ISABIAL), 03010 Alicante, Spain; 2 Cardiology Department, Alicante General University Hospital (HGUA), 03010 Alicante, Spain; 3 Department of Sport Sciences, Sports Research Centre, Miguel Hernández University of Elche, 03202 Elche, Spain; 4 Department of Physical Education and Sport, University of Valencia, 46010 Valencia, Spain; 5 Institute of Biomedicine‑iBiMED and School of Health Sciences, University of Aveiro, 3810-193 Aveiro, Portugal

**Keywords:** Flow-mediated dilation, Endothelial-dependent dilation, Vascular smooth muscle function, Aerobic exercise, HIIT, Exercise modality

## Abstract

**Background:**

Exercise-based cardiac rehabilitation (CR) is considered an effective treatment for enhancing endothelial function in patients with heart failure (HF). However, recent studies have been published and the optimal “dose” of exercise required to increase the benefits of exercise-based CR programmes on endothelial function is still unknown.

**Objectives:**

(a) To estimate the effect of exercise-based CR on endothelial function, assessed by flow-mediated dilation (FMD), in patients with HF; (b) to determine whether high-intensity interval training (HIIT) is better than moderate-intensity training (MIT) for improving FMD; and (c) to investigate the influence of exercise modality (i.e. resistance exercise vs. aerobic exercise and combined exercise vs. aerobic exercise) on the improvement of endothelial function.

**Methods:**

Electronic searches were carried out in PubMed, Embase, and Scopus up to February 2022. Random-effects models of between-group mean differences were estimated. Heterogeneity analyses were performed by means of the chi-square test and *I*^*2*^ index. Subgroup analyses and meta-regressions were used to test the influence of potential moderator variables on the effect of exercise.

**Results:**

We found a FMD increase of 3.09% (95% confidence interval [CI] = 2.01, 4.17) in favour of aerobic-based CR programmes compared with control groups in patients with HF and reduced ejection fraction (HFrEF). However, the results of included studies were inconsistent (*p* < .001; *I*^*2*^ = 95.2%). Higher FMD improvement was found in studies which were randomised, reported radial FMD, or performed higher number of training sessions a week. Moreover, HIIT enhanced FMD to a greater extent than MIT (2.35% [95% CI = 0.49, 4.22]) in patients with HFrEF. Insufficient data prevented pooled analyses for the effect of exercise in patients with HF and preserved ejection fraction and the influence of exercise modality on the improvement of endothelial function.

**Conclusion:**

Aerobic-based CR is a non-pharmacological treatment for enhancing endothelial function in patients with HFrEF. However, higher training frequency and HIIT induce greater adaptation of endothelial function in these patients, which should betaken into consideration when designing exercise-based CR programmes.

*Trial registration* The protocol was prospectively registered on the PROSPERO database (CRD42022304687).

**Supplementary Information:**

The online version contains supplementary material available at 10.1186/s40798-023-00553-z.

## Key Points

- Aerobic-based cardiac rehabilitation (CR) enhances endothelial function in patients with heart failure (HF) and reduced ejection fraction (HFrEF), but the effect of exercise-based CR in patients with HF and preserved ejection fraction requires future study.
- A higher training frequency (e.g., more than two sessions a week) induces systemic vascular adaptations in non-trained limbs and increases the effect of aerobic exercise on endothelial function compared to a low training frequency (e.g., two sessions a week).
- High-intensity interval training increases endothelial function to a greater extent than moderate intensity training in patients with HFrEF.
- Whether resistance exercise or combined exercise is better than aerobic exercise for improving endothelial function should be addressed in future studies.

## Introduction

Heart failure (HF) is a global health pandemic that affects at least 26 million people worldwide, and its prevalence is expected to increase rapidly due to the ageing population [1]. Despite advances in treatment and prevention, mortality and morbidity are still high and the quality of life of these patients is poor [2, 3]. HF is defined as a clinical syndrome characterised by cardinal symptoms (e.g. breathlessness, ankle swelling, and fatigue) that may be accompanied by signs such as pulmonary crackles and peripheral oedema [3]. Exercise intolerance, dyspnea, and/or fatigue are hallmark features of HF [4]. The pathophysiological mechanisms underlying the diminished functional capacity in HF are multifactorial and may differ between patients with reduced ejection fraction (HFrEF) (left ventricular ejection fraction [LVEF] < 50%) and preserved ejection fraction (HFpEF) (LVEF ≥ 50%) [4–7]. These include central cardiac and peripheral mechanisms such as reduced cardiac and pulmonary reserve, skeletal muscle perfusion and/or function, and endothelial dysfunction [4, 6].

Endothelial dysfunction is related to the physiology and progression of HF [8]. It is characterised by an impairment of endothelium-dependent vasodilation caused by a decrease in the bioavailability of vasodilators (e.g. nitric oxide [NO]) and/or an increase in endothelium-derived contracting factors [9]. Endothelial function is commonly assessed by the non-invasive technique of flow-mediated dilation (FMD), which is the dilation of the blood vessels in response to ischemia. FMD is measured by ultrasound, and its primary stimulus is the release of NO from the endothelium due to increased shear stress [10]. Enhanced FMD has been associated with a reduced risk of cardiovascular events in patients with HF [11]. In addition, arterial vasodilation also depends on vascular smooth muscle function. Once NO is synthesised by the endothelial cells, it diffuses into the smooth muscle cells, activating enzymes (e.g. soluble guanylyl cyclase) that are responsible for vessel relaxation [12, 13]. Endothelium-independent nitroglycerin-mediated dilation (NMD) is used to measure the reactivity of the vascular smooth muscle cells via the exogenous administration of NO. Therefore, NMD should be measured complementary to FMD to ensure that decreased FMD values solely reflect endothelial dysfunction and not vascular smooth muscle dysfunction [14].

Exercise-based cardiac rehabilitation (CR) has been shown to improve the prognosis of patients with HF by decreasing morbidity, mortality, and hospital readmissions [15, 16]. The most commonly used exercise modality in CR programmes is aerobic exercise, while resistance exercise and combined aerobic and resistance exercise (henceforth referred to as combined exercise) have been used less frequently [17]. Within aerobic exercise, high-intensity interval training (HIIT) and moderate-intensity training (MIT) are the aerobic exercise methods commonly used [17]. HIIT comprises alternating brief periods of high-intensity aerobic exercise (e.g. > 85% peak oxygen uptake [VO_2_ peak] or above anaerobic threshold) with periods of active (e.g. < 60% VO_2_ peak or below aerobic threshold) or passive recovery of shorter, equal, or longer duration [18]. On the contrary, MIT, which can be carried out continuously or intermittently, is characterised by long-term exercise bouts (e.g. 30 min) performed at moderate intensity (e.g. between aerobic and anaerobic threshold) [19]. Finally, other training variables (e.g. intervention length and training frequency) should also be considered to properly design exercise-based CR programmes.

Previous systematic reviews with meta-analysis have shown the positive effects of exercise-based CR on endothelial function in patients with HF [20, 21]. The enhancement of endothelial function, in turn, has been associated with improved exercise capacity in these patients [22]. However, further evidence has come to light since the publication of these articles [20, 21]. Moreover, their findings showed high inconsistency, and the influence of potential moderator variables (e.g. training frequency) was not analysed. In this regard, Ashor et al. [23] reported a direct association between training frequency and the improvement of endothelial function in healthy people. Regarding other training variables, previous meta-analyses support that HIIT is superior to MIT for improving cardiorespiratory fitness (e.g. VO_2_ peak) and exercise tolerance in patients with HF [24, 25]. Pearson and Smart [21] compared the effect of intermittent exercise with the effect of continuous exercise on endothelial function in patients with HF, but a pure comparison between HIIT and MIT was not included. In addition, the influence of exercise modality on the improvement in endothelial function was not addressed in the previous systematic reviews [20, 21].

Therefore, although exercise-based CR is considered a non-pharmacological tool for enhancing endothelial function in patients with HF, the optimal “dose” of exercise remains unclear, and an update of the literature is required. Hence, the main aims of this systematic review with meta-analysis were: (a) to determine the effect of exercise-based CR on endothelial function (i.e. FMD) in patients with HF; (b) to test whether HIIT enhances endothelial function to a greater degree than MIT; and (c) to investigate the influence of exercise modality (i.e. resistance exercise vs. aerobic exercise and combined exercise vs. aerobic exercise) on the improvement of endothelial function. Secondarily, the effect of exercise-based CR programmes on vascular smooth muscle function (i.e. NMD) was also investigated. Based on previous evidence, we hypothesise that exercise-based CR will enhance endothelial function in patients with HF. Moreover, HIIT will induce a greater increase in endothelial function compared to MIT.

## Methods

The current systematic review with meta-analysis was performed following the Preferred Reporting Items for Systematic Reviews and Meta-Analysis (PRISMA) [26]. The protocol was prospectively registered on the PROSPERO database (CRD42022304687).

### Data Search and Sources

PubMed, Embase, and Scopus were searched from inception to February 2022 to identify potential eligible studies using free-text terms based on the PIO (participants, intervention, and outcomes) strategy (see Additional file 1). The electronic search of each individual database was adapted as necessary. Conference proceedings were also searched on the Web of Science Core Collection. Additionally, previous systematic reviews with and without meta-analysis and references of the studies included in our review were manually screened to identify any additional eligible study. Finally, corresponding authors of included studies were emailed in an attempt to identify ongoing or unpublished additional suitable studies.

### Study Selection

Eligibility criteria were established according to the PICOS (participants, interventions, comparisons, outcomes, and study design) guideline as follows: (a) participants: adult patients with HFrEF or HFpEF regardless of sex. Studies which enrolled HF patients with mechanical assistance (e.g. left ventricular assist devices) and/or heart transplant recipients were excluded; (b) interventions: inpatient or outpatient exercise-based CR programmes lasting at least two weeks, regardless of the setting (i.e. home- or centre-based CR programme), and based mainly on aerobic exercise (i.e. HIIT or MIT), resistance exercise, or combined exercise, either alone or in addition to psychosocial and/or educational interventions; (c) comparisons: (i) exercise-based CR compared to usual care interventions and/or non-exercise groups (henceforth referred to as the control group [CG]), (ii) HIIT compared to MIT, and (iii) resistance exercise compared to aerobic exercise (i.e. HIIT and MIT) or combined exercise compared to aerobic exercise (i.e. HIIT and MIT). Therefore, eligible studies needed to include a CG and/or an intervention group (IG) performing other exercise regimens; (d) outcomes: endothelial and/or vascular smooth muscle function measured noninvasively by FMD (i.e. reactive hyperaemia) and NMD (i.e. sublingual administration), respectively, in either upper (i.e. brachial and radial) and/or lower (i.e. femoral and tibial) limb arteries, and reported as relative (%) and/or absolute (mm) changes; and (e) study design: randomised and non-randomised studies. Finally, studies written in English or Spanish were included. When several articles referred to the same study, the original article was included in the review.

Two authors (C.B. and N.S.) assessed all identified studies for possible inclusion. In case of disagreements, a third author (A.M.) checked the study information to reach an agreement.

### Data Extraction and Coding Study Characteristics

Two authors (C.B. and L.F.) coded the characteristics of the full-text included studies using a standardised extraction form. When there was doubt, a third author (A.M.) assessed the information to reach an agreement.

The information extracted from the studies was classified as follows: (a) study characteristics (publication year, country, journal, and study design [i.e. randomised or non-randomised]); (b) patient characteristics (sample size, sex [i.e. males, females, or mixed sample], men percentage, age, baseline artery diameter [mm], LVEF, VO_2_ peak, New York Heart Association [NYHA] functional class, and implantable cardioverter defibrillator); (c) intervention characteristics (CR phase [i.e. inpatient or outpatient], setting [i.e. home- or centre-based CR programme], exercise modality [i.e. aerobic exercise, resistance exercise, or combined exercise], aerobic exercise method (if applicable) [i.e. HIIT or MIT], intervention length [weeks], sessions a week, and intervention description [e.g. session length, intensity, training mode, and number of sets]); (d) CG details (instructions given to patients and activity monitoring); (e) FMD assessment characteristics (artery measured, cuff placement [i.e. distal or proximal to the imaged artery], occlusion length [seconds], occlusion pressure [mmHg], and post-deflation time window [seconds]); (f) NMD assessment characteristics (artery measured, dose [mg], and post-administration time window [seconds]); and (g) statistical information (e.g. mean and standard deviation [*SD*]).

### Dealing with Missing Data

Corresponding authors were emailed to request key study characteristics (e.g. patient and intervention characteristics) and obtain missing numerical outcome data when a study was identified as “abstract only” or outcome data were not reported or presented graphically, respectively. If no response was received, abstracts were excluded from the review and outcome data were extracted from the figures.

### Methodological Quality Assessment

The tool for the assessment of study quality and reporting in exercise (TESTEX) scale was used to carry out the methodological quality judgement of the included studies [27]. The TESTEX scale consists of 12 items, which are answered with “yes” (1 point) or “no” (0 points) if the criterion is satisfied or not, respectively, and the maximum number of points is 15. The criteria used to carry out methodological quality assessment can be found in Additional file 1: Table S1. Based on the overall scores, methodological quality was judged as excellent (12–15), good (9–11), fair (6–8), or poor (< 6). Two authors (A.M. and L.F.) carried out the methodological quality assessment and, in case of doubts, a third author (J.M.S) checked the specific item to reach an agreement.

### Computation of Effect Size and Statistical Analyses

The mean difference (MD) with its 95% confidence interval (CI) was used as the effect size (ES) index. The MD was calculated by subtracting the mean change in the comparison group (i.e. CG, MIT, and aerobic exercise) from the mean change in the reference group (i.e. exercise-based CR, HIIT, resistance exercise, or combined exercise) and was then corrected by a factor for small samples [28]. When a study included more than one IG and a shared CG, the sample size of the CG was split by the number of IGs to avoid overinflation of the sample size [29], allowing us to include several analysis units from the same study in the same pooled analysis. Separate meta-analyses were performed based on the outcome (i.e. FMD or NMD) and unit of measurement (i.e. relative [%] or absolute [mm] changes). Moreover, upper limb arteries (i.e. brachial and radial) were selected as preferential measurements for studies which assessed endothelial function in upper and lower limb arteries. A random-effects model was used to conduct pooled analyses, in which the weighting factor is the inverse variance, defined as the sum of the within-study and the between-study variance [30]. Meta-analysis was performed only if three or more analysis units were included for the specific endpoint.

Regarding heterogeneity, the chi-square test was used to identify statistical heterogeneity and the *I*^*2*^ index was used to quantify the percentage of variation across studies due to heterogeneity. *I*^*2*^ values of 25%, 50%, and 75% were interpreted as low, moderate, and high heterogeneity, respectively [31]. Statistical (*p* ≤ 0.050) and/or moderate–high heterogeneity (*I*^*2*^ ≥ 50%) was considered as indicative of substantial heterogeneity [31], and therefore, heterogeneity analyses were performed. The influence of potential moderator variables on the change of endothelial function was analysed by means of subgroup analyses for categorical variables (i.e. study design [i.e. randomised vs. non-randomised], artery [i.e. brachial vs. radial], sex, aerobic exercise method [i.e. HIIT vs. MIT], and sessions a week [i.e. > 3 sessions vs. ≤ 3 sessions]), and simple meta-regressions for continuous variables (i.e. intervention length, sessions a week, and total number of exercise sessions). All analyses were performed using weighted least squares and assuming mixed-effect models [32]. Heterogeneity analyses were performed if a minimum of 10 analysis units were meta-analysed.

Sensitivity analyses were performed to test the robustness of our findings as follows: (a) applying the leave-one-out cross-validation method, which consists of carrying out the pooled analysis on each subset of the included studies by leaving out one study at a time; (b) removing studies whose methodological quality in the TESTEX scale was judged as poor (< 6); and (c) applying robust variance estimation, which has been proposed to handle dependent ES in meta-analysis [33, 34]. Specifically, robust variance estimation allows to control dependent outcomes reported by the same research group in different publications [35]. Thus, the research group was used as the clustering variable to carry out sensitivity analysis. Additionally, small-study effect was assessed for publication bias control. In this regard, the publication bias was analysed graphically through contour-enhanced funnel plots, while the Egger’s test was used to quantify the evidence for funnel plot asymmetry [36, 37]. Sensitivity and publication bias analyses were carried out if at least 10 analysis units were included in the pooled analysis [37]. All analyses were performed using STATA software (version 16.0; Stata Corp LLC, College Station, TX, USA).

### Deviations from Registered Protocol

Regarding heterogeneity analyses, the imaged artery used to carry out endothelial function assessment had not been included as a potential moderator variable. Moreover, after data extraction, training frequency was included as a categorical variable. Finally, we also found several studies performed by the same research group. Thus, robust variance estimation was also used to carry out sensitivity analyses.

## Results

### Study Selection

**Fig. 1:**
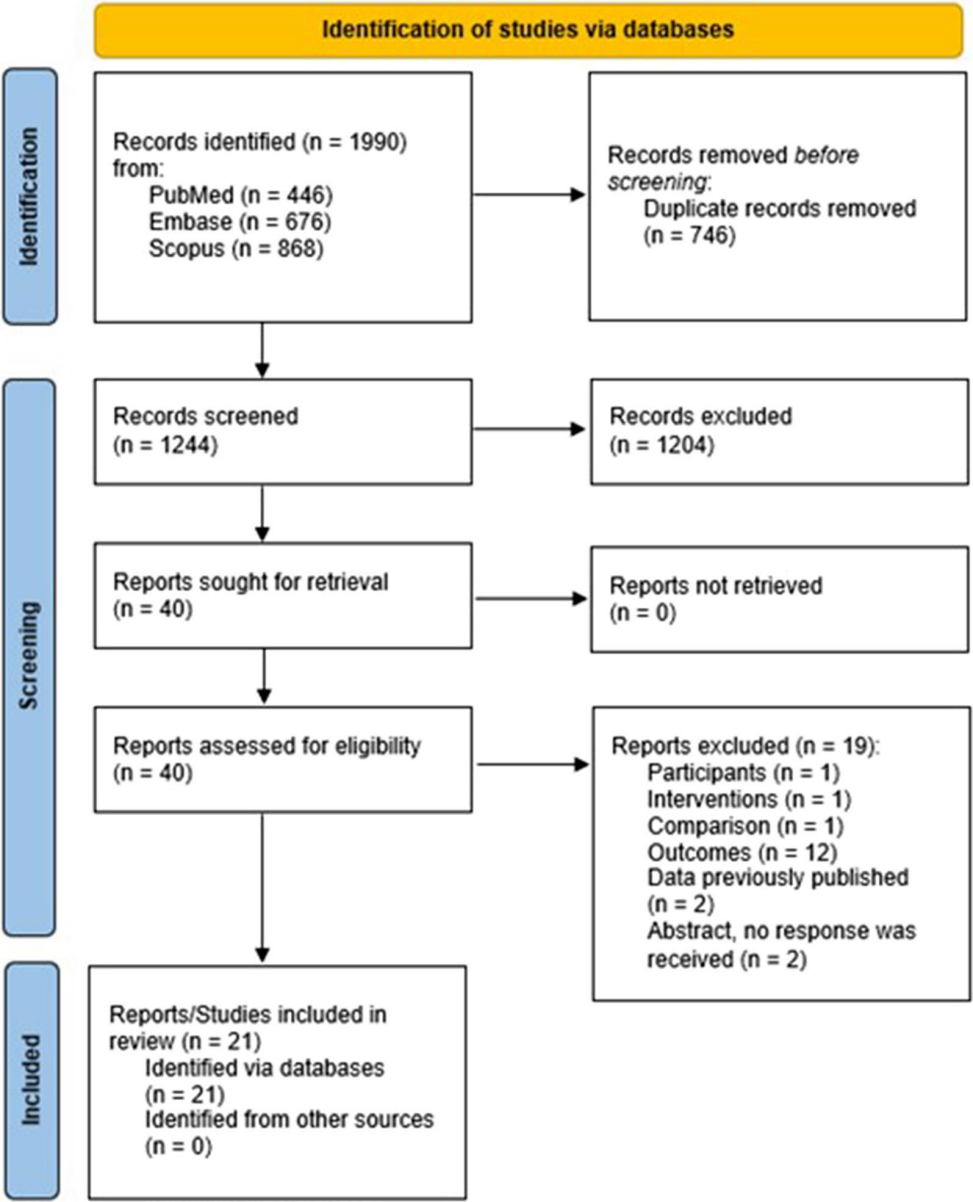
Flow chart of the study selection process

The study selection process is presented in Fig. 1. Briefly, the electronic database search retrieved 1244 records after removing duplicates (*n* = 746). After reviewing titles and abstracts, 40 studies were eligible for full-text analysis, of which 21 were included [38–58] in the qualitative synthesis and 19 were excluded (see Fig. 1 for reasons). No additional studies were identified from other sources. Although efforts were made to identify unpublished studies, all studies included in this review had been published in peer-reviewed journals.

### Study Characteristics

Study and patient characteristics can be found in Table 1. Out of all the 21 selected studies, 12 (57%) included one IG and one CG [40–42, 44–51, 56], four (19%) included two IGs and one CG [43, 53, 55, 57], four (19%) included two IGs [38, 39, 52, 58], and one (5%) included two IGs and two CGs based on patient’s age [54]. Thus, 30 IGs and 18 CGs were defined in this systematic review. The 21 studies were published from 2001 to 2021. Eighteen studies (86%) were randomised [38–42, 44–47, 49–55, 57, 58], and three (14%) were non-randomised [43, 48, 56]. The 21 studies enrolled 738 patients, 457 in the IGs and 281 in the CGs. The sample size in the IGs ranged from five to 44 patients, with a mean ± *SD* age of 60.8 ± 7.3 years (min to max: 50.0 to 76.5 years), and the sample size in the CGs varied from four to 42 patients, with a mean ± *SD* age of 61.6 ± 7.3 years (min to max: 49.0 to 75.5 years). Fourteen studies (67%) recruited male and female patients [38, 39, 42, 43, 46, 48–50, 52–57], and seven (33%) recruited exclusively male patients [40, 41, 44, 45, 47, 51, 58]. Eighteen studies (86%) enrolled patients with HFrEF [38, 40–48, 50–54, 56–58], two (9%) recruited patients with HFpEF [39, 49], and one (5%) enrolled patients with HFrEF and HFpEF [55]. Four studies (19%) explicitly disclosed that patients with implantable cardioverter defibrillators had been recruited [40, 45, 48, 54]. Eighteen studies (86%) enrolled patients with different NYHA functional class (e.g. II and III) [38–43, 46–56, 58], two (9%) recruited exclusively patients with NYHA II [44] and III [45], and one (5%) did not disclose this information [57].

**Table 1:** Study and patient characteristics

Study	Group; EM (AEM)	Study characteristics	Patient characteristics	
		Country; study design; journal	Sample size (analysed); male percentage; age; baseline diameter	LVEF; VO_2_ peak; NYHA
Anagnostakou et al. [38]	IG; CE (HIIT)	Greece; randomised; J Card Fail	14 (14); 79%; 54.0 ± 10.0 y; 4.50 ± 0.50 mm (brachial)	39.0 ± 11.0%; 15.7 ± 6.0 ml·kg^−1^·min^−1^; 5 (I), 7 (II), and 2 (III)
	IG^$^; AE (HIIT)		14 (14); 86%; 52.0 ± 11.0 y; 4.40 ± 0.50 mm (brachial)	36.0 ± 13.0%; 15.7 ± 4.0 ml·kg^−1^·min^−1^; 3 (I), 10 (II), and 1 (III)
Angadi et al. [39]	IG; AE (HIIT)	USA; randomised; J Appl Physiol	9 (9); 89%; 69.0 ± 6.1 y; NR (brachial)	65.0 ± 5.0%*; 19.2 ± 5.2 ml·kg^−1^·min^−1^; II-III (IC)
	IG^$^; AE (MIT)		6 (6); 67%; 71.5 ± 11.7 y; NR (brachial)	66.0 ± 4.0%*; 16.9 ± 3.0 ml·kg^−1^·min^−1^; II-III (IC)
Belardinelli et al. [40]	IG; AE (MIT)	Italy; randomised; Eur J Cardiovasc Prev Rehabil	30 (30); 100%; 55.1 ± 14.0 y; NR (brachial)	30.2 ± 7.0%; 14.8 ± 2.5 ml·kg^−1^·min^−1^; 17 (II) and 13 (III)
	CG; NA		22 (22); 100%; 53.1 ± 15.0 y; NR (brachial)	33.6 ± 8.0%; 15.8 ± 2.1 ml·kg^−1^·min^−1^; 12 (II) and 10 (III)
Belardinelli et al. [41]	IG; AE (MIT)	Italy; randomised; Int J Cardiol	30 (30); 100%; 55.9 ± 15.0 y; NR (brachial)	29.3 ± 6.0%; 16.8 ± 3.7 ml·kg^−1^·min^−1^; 18 (II) and 12 (III)
	CG; NA		29 (29); 100%; 58.0 ± 12.0 y; NR (brachial)	28.1 ± 5.0%; 15.9 ± 1.5 ml·kg^−1^·min^−1^; 15 (II) and 14 (III)
Belardinelli et al. [42]	IG; AE (MIT)	Italy; randomised; Circ Heart Fail	44 (44); 86%; 59.0 ± 10.0 y; NR (brachial)	35.0 ± 8.0%; 16.5 ± 4.5 ml·kg^−1^·min^−1^; 31 (II) and 13 (III)
	CG; NA		42 (42); 83%; 58.0 ± 10.0 y; NR (brachial)	37.0 ± 8.0%; 16.1 ± 4.5 ml·kg^−1^·min^−1^; 29 (II) and 13 (III)
Benda et al. [43]	IG; AE (HIIT)	Netherlands; non-randomised; PloS One	10 (10); 90%; 63.0 ± 8.0 y; 4.40 ± 0.90 mm (brachial) 6.70 ± 1.10 mm (femoral)	37.0 ± 6.0%; 19.1 ± 4.1 ml·kg^−1^·min^−1^; 8 (II) and 2 (III)
	IG^$^; AE (MIT)		10 (10); 100%; 64.0 ± 8.0 y; 4.50 ± 0.50 mm (brachial) 7.10 ± 1.20 mm (femoral)	38.0 ± 6.0%; 21.0 ± 3.4 ml·kg^−1^·min^−1^; 8 (II) and 2 (III)
	CG; NA		9 (9); 56%; 67.0 ± 7.0 y; 4.10 ± 0.80 mm (brachial) 6.20 ± 1.00 mm (femoral)	40.0 ± 11.0%; 17.4 ± 5.8 ml·kg^−1^·min^−1^; 8 (II) and 1 (III)
Eleuteri et al. [44]	IG; AE (MIT)	Italy; randomised; Biomarkers	11 (11); 100%; 66.0 ± 6.6 y; 4.40 ± 0.30 mm (brachial)	28.0 ± 7.0%; 14.8 ± 2.3 ml·kg^−1^·min^−1^; 11 (II)
	CG; NA		10 (10); 100%; 63.0 ± 6.3 y; 4.40 ± 0.30 mm (brachial)	30.0 ± 5.7%; 16.7 ± 1.3 ml·kg^−1^·min^−1^; 10 (II)
Erbs et al. [45]	IG; AE (MIT)	Germany; randomised; Circ Heart Fail	18 (17); 100%; 60.0 ± 11.0 y; NR (radial)	24.1 ± 5.1%; 15.3 ± 3.3 ml·kg^−1^·min^−1^; 18 (III)
	CG; NA		19 (17); 100%; 62.0 ± 10.0 y; NR (radial)	25.0 ± 4.3%; 15.4 ± 3.8 ml·kg^−1^·min^−1^; 19 (III)
Giannattasio et al. [46]	IG; AE (MIT)	Italy; randomised; Hypertension	11 (11); NR (MS); NR; 2.90 ± 0.33 mm (radial)	32.9 ± 11.3%; NR; NR (I), NR (II), and NR (III)
	CG; NA		11 (11); NR (MS); NR; 2.50 ± 0.33 mm (radial)	32.2 ± 2.3%; NR; NR (I), NR (II), and NR (III)
Guazzi et al. [47]	IG; AE (MIT)	Italy; randomised; J Appl Physiol	16 (16); 100%; 52.0 ± 5.0 y; NR (brachial)	34.3 ± 3.3%; 17.1 ± 2.8 ml·kg^−1^·min^−1^; 11 (II) and 5 (III)
	CG; NA		15 (15); 100%; 54.0 ± 4.0 y; NR (brachial)	35.5 ± 3.7%; 16.5 ± 2.7 ml·kg^−1^·min^−1^; 10 (II) and 5 (III)
Isaksen et al. (48)	IG; AE (HIIT)	Norway; non-randomised; Eur J Prev Cardiol	24 (24); 88%; 65.0 ± 9.0 y; 3.63 ± 0.82 mm (brachial)	37.6 ± 10.9%; 17.4 ± 4.6 ml·kg^−1^·min^−1^; 4 (I), 18 (II), and 2 (III)
	CG; NA		11 (11); 100%; 69.0 ± 9.0 y; 3.94 ± 0.61 mm (brachial)	30.0 ± 8.1%; 16.9 ± 2.8 ml·kg^−1^·min^−1^; 11 (II)
Kitzman et al. [49]	IG; AE (MIT)	USA; randomised; J Am Coll Cardiol	32 (23); 28%; 70.0 ± 7.0 y; 4.17 ± 0.91 mm (brachial)	58.0 ± 6.0%*; 14.2 ± 2.8 ml·kg^−1^·min^−1^; 15 (II) and 17 (III)
	CG; NA		31 (22); 20%; 70.0 ± 7.0 y; 4.13 ± 0.87 mm (brachial)	56.0 ± 5.0%*; 14.0 ± 3.2 ml·kg^−1^·min^−1^; 17 (II) and 14 (III)
Kobayashi et al. [50]	IG; AE (MIT)	Japan; randomised; Circ J	14 (14); 86%; 55.0 ± 7.5 y; 4.53 ± 0.64 mm (brachial) 2.76 ± 0.60 mm (tibial)	29.0 ± 7.5%; 18.0 ± 4.9 ml·kg^−1^·min^−1^; 10 (II) and 4 (III)
	CG; NA		14 (14); 57%; 62.0 ± 7.5 y; 4.16 ± 0.82 mm (brachial) 2.66 ± 0.52 mm (tibial)	33.0 ± 7.5%; 13.7 ± 3.4 ml·kg^−1^·min^−1^; 8 (II) and 6 (III)
Linke et al. [51]	IG; AE (MIT)	Germany; randomised; J Am Coll Cardiol	11 (11); 100%; 58.0 ± 6.6 y; 3.42 ± 0.12 mm (radial)	26.0 ± 9.9%; 16.0 ± 4.0 ml·kg^−1^·min^−1^; 8 (II) and 3 (III)
	CG; NA		11 (11); 100%; 59.0 ± 10.0 y; 2.99 ± 0.13 mm (radial)	24.0 ± 6.6%; 16.9 ± 4.3 ml·kg^−1^·min^−1^; 8 (II) and 3 (III)
Munch et al. [52]	IG; RE (NA)	Denmark; randomised; J Cardiopulm Rehabil Prev	12 (12); 83%; 59.0 ± 10.4 y; 5.99 ± 0.90 mm (femoral)	≤ 40% (IC); 23.0 ± 3.5 ml·kg^−1^·min^−1^; 2 (I) and 10 (II)
	IG^$^;AE (MIT)		14 (13); 86%; 63.0 ± 11.2 y; 6.17 ± 1.01 mm (femoral)	≤ 40% (IC); 22.0 ± 3.7 ml·kg^−1^·min^−1^; 4 (I) and 10 (II)
Sales et al. [53]	IG; AE (HIIT)	Brazil; randomised; Circ Heart Fail	11 (9); 64%; 55.0 ± 7.6 y; 4.20 ± 0.50 mm (brachial)	27.8 ± 9.5%; 17.9 ± 3.2 ml·kg^−1^·min^−1^; 6 (II) and 5 (III)
	IG^$^; AE (MIT)		11 (10); 64%; 59.5 ± 7.0 y; 4.00 ± 0.80 mm (brachial)	31.3 ± 6.1%; 16.9 ± 1.9 ml·kg^−1^·min^−1^; 9 (II) and 2 (III)
	CG; NA		8 (8); 75%; 56.1 ± 7.0 y; 4.20 ± 0.70 mm (brachial)	25.8 ± 8.1%; 15.7 ± 3.5 ml·kg^−1^·min^−1^; 4 (II) and 4 (III)
Sandri et al. [54]	≤ 55 y: IG; AE (MIT)	Germany; randomised; Eur J Prev Cardiol	15 (15); 80%; 50.0 ± 5.0 y; NR (radial)	27.0 ± 6.0%; 13.3 ± 1.6 ml·kg^−1^·min^−1^; 8 (II) and 7 (III)
	≤ 55 y: CG; NA		15 (15); 87%; 49.0 ± 5.0 y; NR (radial)	28.0 ± 5.0%; 13.6 ± 1.3 ml·kg^−1^·min^−1^; 9 (II) and 6 (III)
	≥ 65 y: IG; AE (MIT)		15 (15); 80%; 72.0 ± 4.0 y; NR (radial)	29.0 ± 6.0%; 12.9 ± 1.4 ml·kg^−1^·min^−1^; 7 (II) and 8 (III)
	≥ 65 y: CG; NA		15 (15); 80%; 72.0 ± 3.0 y; NR (radial)	28.0 ± 6.0%; 13.1 ± 1.5 ml·kg^−1^·min^−1^; 8 (II) and 7 (III)
Turri-Silva et al. [55]	IG; RE (NA)	Norway; randomised; PloS One	6 (6); 67%; 55.0 ± 10.9 y; 4.38 ± 0.58 mm (brachial)	42.2 ± 13.5%*; 16.9 ± 2.5 ml·kg^−1^·min^−1^; 2 (I), 3 (II), and 1 (III)
	IG^$^; AE (HIIT)		8 (5); 63%; 60.9 ± 9.7 y; 4.54 ± 1.09 mm (brachial)	50.4 ± 17.0%*; 17.5 ± 4.2 ml·kg^−1^·min^−1^; 3 (I), 3 (II), and 2 (III)
	CG; NA		8 (4); 88%; 56.0 ± 9.7 y; 4.36 ± 1.17 mm (brachial)	46.8 ± 14.4%*; 16.9 ± 2.5 ml·kg^−1^·min^−1^; 5 (I), 2 (II), and 1 (III)
Van Craenenbroeck et al. [56]	IG; AE (MIT)	Belgium; non-randomised; Basic Res Cardiol	21 (21); 86%; 61.3 ± 10.1 y; 4.81 ± 0.14 mm (brachial)	27.0 ± 8.7%; 18.3 ± 6.4 ml·kg^−1^·min^−1^; NR (2.3 ± 0.5)
	CG; NA		17 (17); 71%; 63.4 ± 12.4 y; 4.50 ± 0.50 mm (brachial)	31.3 ± 7.0%; 21.3 ± 8.7 ml·kg^−1^·min^−1^; NR (2.0 ± 0.4)
Wisløff et al. [57]	IG; AE (HIIT)	Norway; randomised; Circulation	9 (9); 78%; 76.5 ± 9.0 y; NR (brachial)	28.0 ± 7.3%; 13.0 ± 1.6 ml·kg^−1^·min^−1^; NR
	IG^$^; AE (MIT)		8 (8); 78%; 74.4 ± 12.0 y; NR (brachial)	32.8 ± 4.8%; 13.0 ± 1.1 ml·kg^−1^·min^−1^; NR
	CG; NA		9 (9); 67%; 75.5 ± 13.0 y; NR (brachial)	26.2 ± 8.0%; 13.2 ± 1.9 ml·kg^−1^·min^−1^; NR
Zaky et al. [58]	IG; AE (HIIT)	Egypt; randomised; Biosci Res	20 (20); 100%; 54.0 ± 2.7 y; 4.13 ± 0.26 mm (brachial)	37.0 ± 1.9%; NR; II-III (IC)
	IG^$^;AE (MIT)		20 (20); 100%; 52.8 ± 11.6 y; 4.13 ± 0.27 mm (brachial)	37.5 ± 3.1%; NR; II-III (IC)

AE, aerobic exercise; AEM, aerobic exercise method; CE, combined aerobic and resistance exercise; CG, control group; EM, exercise modality; HIIT, high-intensity interval training; IC, inclusion criterion; IG, intervention group; LVEF, left ventricular ejection fraction; MIT, moderate-intensity training; MS, mixed sample; NA, not applicable; NYHA, New York Heart Association functional class; NR, not reported; RE, resistance exercise; VO_2_ peak, peak oxygen uptake; and y, years

Values are reported as mean ± standard deviation

^$^The intervention group was also considered the comparator group

^*^Patients with preserved left ventricular ejection fraction (> 50%) were included

Intervention and assessment characteristics are summarised in Additional file 1: Table S2. Out of all the 21 studies included, 17 (81%) performed a centre-based CR programme [38–43, 47–56, 58], one (5%) carried out a home-based CR programme [44], two (9%) combined centre- and home-based exercise sessions [45, 57], and one (5%) did not report this information [46]. Two studies (9%) carried out an inpatient CR programme [51, 54]. The number of exercise sessions performed a week varied from two to eight sessions, while the intervention duration ranged from four to 24 weeks. The total number of exercise sessions ranged from 12 to 96 sessions. Out of all the 30 IGs, 27 (90%) used aerobic exercise as the exercise modality, two (7%) performed resistance exercise, and one (3%) carried out combined exercise. Out of all the 28 IGs which performed aerobic exercise (alone or combined with resistance exercise), 19 (68%) used MIT as the aerobic exercise method and nine (32%) used HIIT. Details of the intervention characteristics (e.g. exercise mode [e.g. cycle ergometer], sessions length, and intensity) can be found in Additional file 1: Table S2.

Regarding assessment characteristics, all the included studies (100%) assessed endothelial function [38–58] and seven (33%) also measured vascular smooth muscle function [40, 41, 43, 46–48, 57]. Fourteen studies (67%) measured endothelial function in the brachial artery [38–42, 44, 47–49, 53, 55–58], four (19%) in the radial artery [45, 46, 51, 54], one (5%) in the femoral artery [52], and two (9%) in the brachial artery and lower limb arteries (i.e. femoral and posterior tibial) [43, 50]. Eleven studies (52%) placed the cuff distal to the appraised artery [38, 40–44, 46, 47, 49, 53, 56], five (24%) proximal [45, 51, 54, 55, 58], and one (5%) positioned the cuff distal to the brachial artery and proximal to the posterior tibial artery [50], while four (19%) did not specifically disclose this information [39, 48, 52, 57] and guidelines for measuring FMD were referenced [14, 59]. The remaining details of the assessment characteristics (i.e. occlusion length, occlusion pressure, post-deflation time window, nitroglycerin dose, and post-administration time window) can be found in Additional file 1: Table S2. Five studies (24%) reported their findings in relative and absolute values [38, 48, 49, 51, 53], 15 (71%) in relative values [39–45, 47, 50, 52, 54–58], and one (5%) in absolute values [46].

### Methodological Quality Assessment

A summary of the methodological quality assessment using the TESTEX scale is shown in Additional file 1: Table S3. The mean ± *SD* TESTEX score was 7.4 ± 2.3 (min to max: three to 12). Reviewers deemed four studies (19%) to have poor quality [44, 46, 53, 58], 11 (52%) to have fair quality [38–40, 43, 47–49, 51, 55–57], five (24%) to have good quality [41, 42, 45, 50, 52], and one (5%) to have excellent quality [54]. The noteworthy findings from the methodological quality assessment showed that, out of the 18 randomised studies, 11 (61%) and 12 (67%) failed to report specific details of the random sequence generation and allocation concealment, respectively. Out of the 21 included studies, nine (43%) did not blind assessors of endothelial function to patient’s allocation. Twelve studies (57%) failed to clearly report pre- and post-intervention sample sizes, or the withdrawal rate was higher than 15%. Three studies (14%) clearly disclosed that all patients completed the intervention period (no dropouts). None of the included studies performed intention-to-treat analysis or reported physical activity data of the CG. Out of the 18 studies which carried out an intervention with a duration longer than four weeks, only one (6%) performed a mid-intervention assessment.

### Outcome Measures

#### Training-Induced Effect on Endothelial Function

Five studies [38, 39, 49, 52, 55], which included patients with HFpEF (*n* = 3) [39, 49, 55] and/or investigated the effect of resistance exercise (*n* = 2) [52, 55] or combined exercise (*n* = 1) [38], were excluded from meta-analyses due to the low number of trials included in the systematic review. Their findings will be qualitatively analysed in the Discussion section. Therefore, the following pooled findings refer to the effect of aerobic exercise in patients with HFrEF.

**Fig. 2:**
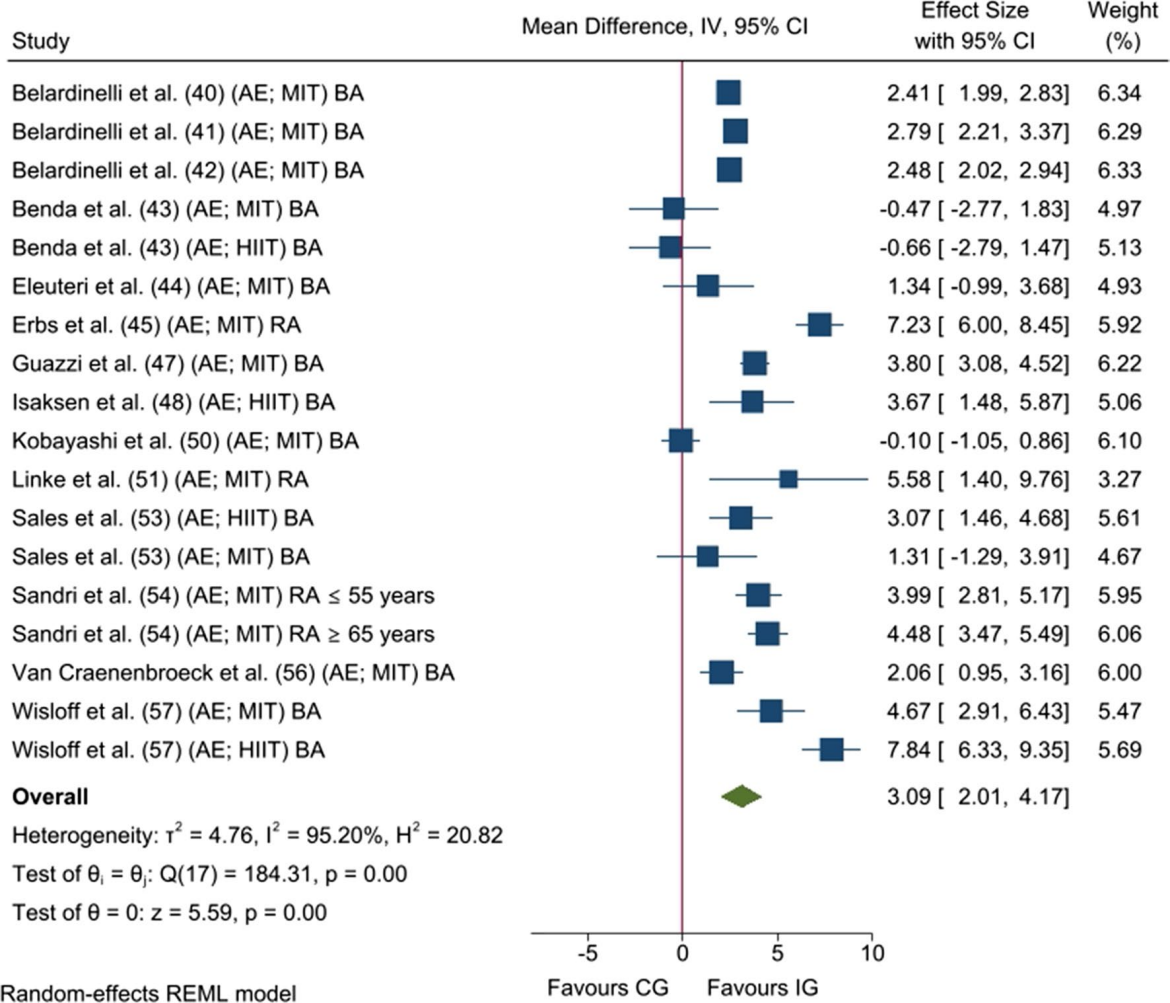
Forest plot of mean differences between exercise-based cardiac rehabilitation and control group for relative flow-mediated dilation. AE, aerobic exercise; BA, brachial artery; CG, control group; CI, confidence interval; HIIT, high-intensity interval training; IG, intervention group; IV, inverse variance; MIT, moderate-intensity training; and RA, radial artery

Meta-analysed data from 14 studies (18 analysis units; 305 [IG] and 244 [CG] patients) revealed a statistically significant improvement in relative FMD values (*p* < 0.001; MD_+_  = 3.09% [95% CI = 2.01, 4.17]; Fig. 2) after an aerobic-based CR programme compared with CG. The heterogeneity test reached statistical significance (*p* < 0.001), and inconsistency was high (*I*^*2*^ = 95.2%).

Analyses of the influence of potential moderator variables on the improvement of relative FMD can be found in Additional file 1: Table S4. Subgroup analyses reached statistical significance for the study design (*p* = 0.040), type of artery (*p* = 0.008), and sessions a week (*p* = 0.050). When taking these variables into consideration, results showed higher endothelial function improvement in randomised trials (14 analysis units; MD_+_  = 3.59% [95% CI = 2.43, 4.76]; *I*^*2*^ = 95.5%) compared with non-randomised trials (four analysis units; MD_+_  = 1.21% [95% CI = –0.74, 3.17]; *I*^*2*^ = 77.0%), studies which measured radial FMD (four analysis units; MD_+_  = 5.25% [95% CI = 3.58, 6.92]; *I*^*2*^ = 81.5%) compared with those that assessed brachial FMD (14 analysis units; MD_+_  = 2.51% [95% CI = 1.36, 3.65]; *I*^*2*^ = 95.0%), and in studies which performed > 3 sessions a week (six analysis units; MD_+_  = 4.41% [95% CI = 2.88, 5.94]; *I*^*2*^ = 87.8%) compared with those that carried out ≤ 3 sessions (12 analysis units; MD_+_  = 2.46% [95% CI = 1.15, 3.78]; *I*^*2*^ = 95.7%). Simple meta-regressions also showed a direct relationship between relative FMD enhancement and sessions a week (18 analysis units; *B* = 0.82% [95% CI = 0.29, 1.25]; *p* = 0.002).

**Fig. 3:**
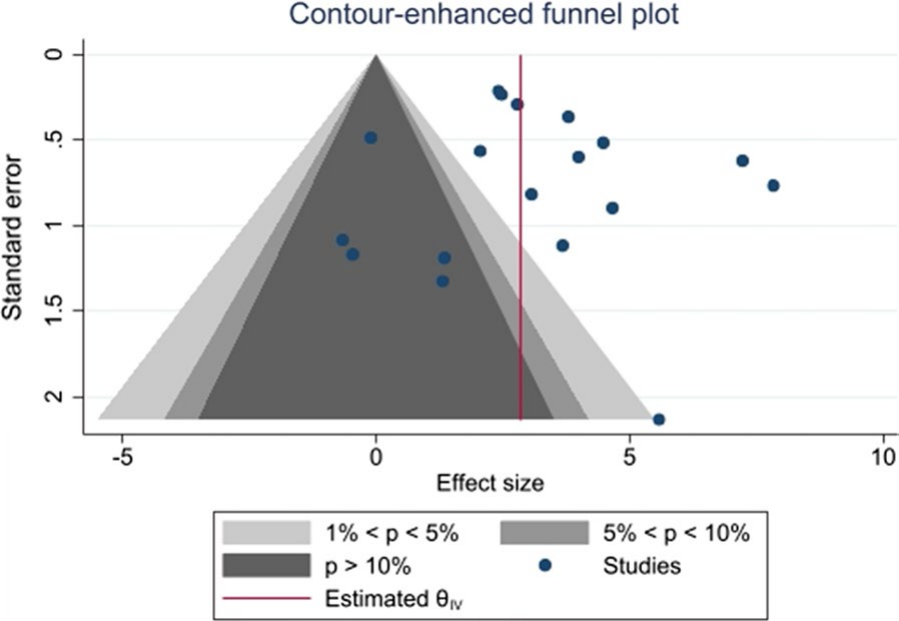
Contour-enhanced funnel plot for relative flow-mediated dilation

Regarding sensitivity analyses, the conclusions were similar to those obtained before using the leave-one-out cross-validation method and excluding analysis units reported from studies whose methodological quality was judged as poor. Finally, although the conclusions were similar to those obtained before running the random variance estimation analysis, the magnitude of the pooled MD slightly decreased (*p* = 0.004; MD_+_  = 2.64% [95% CI = 1.12, 4.16]) when compared with the former analysis (see Fig. 2). The Egger's test revealed no small-study effect (*p* = 0.851). In addition, the contour-enhanced funnel plot shows no asymmetry (Fig. 3), and no studies appear to be missing in the non-significant areas [37]. Therefore, publication bias can be discarded as a threat against our finding regarding the effect of aerobic exercise on relative FMD.

Combined data from four studies (five analysis units; 65 [IG] and 41 [CG] patients) showed a statistically significant enhancement in absolute FMD values (*p* < 0.001; MD_+_  = 0.13 mm [95% CI = 0.10, 0.16]; Additional file 1: Fig. S1) after an aerobic-based CR programme compared to CG. The heterogeneity test did not reach statistical significance (*p* = 0.634), and no inconsistency was found (*I*^*2*^ = 0.0%).

**Fig. 4:**
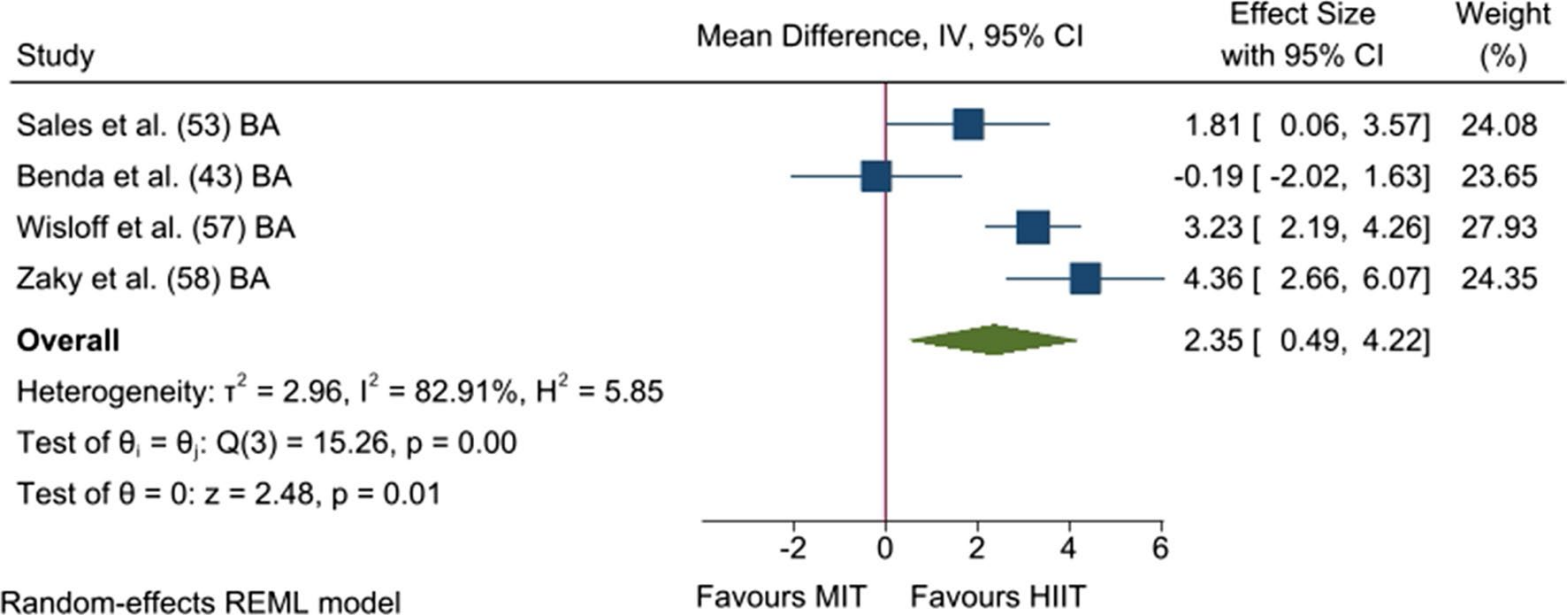
Forest plot of mean differences between high-intensity interval training and moderate-intensity training for relative flow-mediated dilation. BA, brachial artery; CI, confidence interval; HIIT, high-intensity interval training; IV, inverse variance; and MIT, moderate-intensity training

Regarding the aerobic exercise method, meta-analysed data from four studies (four analysis units; 48 [HIIT] and 48 [MIT] patients) revealed that HIIT increased relative FMD values to a higher extent than MCT (*p* = 0.013; MD_+_  = 2.35% [95% CI = 0.49, 4.22]; Fig. 4). The heterogeneity test reached statistical significance (*p* = 0.002), and inconsistency was high (*I*^*2*^ = 82.9%).

#### Training-Induced Effect on Vascular Smooth Muscle Function

Two studies did not report enough information to calculate ES and were excluded from meta-analysis [46, 57]. Pooled data from five studies (six analysis units; 119 [IG] and 86 [CG] patients) showed that aerobic-based CR did not enhance relative NMD to a greater extent than CG (*p* = 0.424; MD_+_  = 0.29% [95% CI = − 0.43, 1.01]; Additional file 1: Fig. S2). The heterogeneity test reached statistical significance (*p* = 0.008), and inconsistency was moderate (*I*^*2*^ = 60.5%).

## Discussion

This systematic review with meta-analysis was conducted to investigate the effect of exercise-based CR programmes on endothelial function (i.e. FMD) in patients with HFrEF and HFpEF, as well as to assess the influence of the aerobic exercise method (i.e. HIIT vs. MIT) and exercise modality (i.e. resistance exercise vs. aerobic exercise and combined exercise vs. aerobic exercise) on the improvement of endothelial function. Additionally, the effect of exercise-based CR on vascular smooth muscle function (i.e. NMD) was also investigated. However, the low number of studies prevented pooled analyses for studying the effect of exercise-based CR in patients with HFpEF and for investigating the influence of exercise modality on the improvement of endothelial function. Our main finding showed that aerobic-based CR programmes enhance FMD in patients with HFrEF, supporting the idea that exercise training is a non-pharmacological therapy capable of restoring endothelial function in these patients. Moreover, HIIT enhances endothelial function to a greater extent than MIT in these patients. Finally, aerobic-based CR did not enhance vascular smooth muscle function in patients with HFrEF.

### Training-Induced Effect on Endothelial Function

In accordance with our hypothesis, we found that aerobic-based CR is a non-pharmacological treatment for enhancing endothelial function in patients with HFrEF. Several mechanisms have been used to explain the effect of aerobic exercise on endothelial function in patients with HF. First, aerobic exercise-induced shear stress increases NO availability [60–62] by inducing endothelial NO synthase phosphorylation [63]. Additionally, exercise training promotes vascular healing and neovascularisation by inducing endothelial progenitor cells mobilisation from the bone marrow to the circulation [64–66], which is mediated by pro-angiogenic factors such as chemokines (e.g. stromal cell derived factor 1 alpha), growth factors (e.g. vascular endothelial growth factors), and cytokines (i.e. interleukin-8) [67]. Finally, exercise training also increases endothelium-reparative capacity by enhancing intracellular signalling [68].

Our results showed an increase of 3.09% (95% CI = 2.01, 4.17) in relative FMD after an aerobic-based CR programme compared to usual care and/or non-exercise. We also found an improvement in endothelial function when we used absolute FMD values (0.13 mm [95% CI = 0.10, 0.16]). These data are in line with previous systematic reviews and meta-analyses of patients with HF [69]. For instance, Pearson and Smart [21] showed a statistically significant increase in relative FMD after moderate- and vigorous-intensity aerobic exercise (1.00 [95% CI = 0.19, 1.80], and 1.21 [95% CI = 0.60, 1.82], respectively). Moreover, Pearson and Smart [20], who included other types of intervention (i.e. yoga, pilates, tai chi, hydrotherapy, functional electrical stimulation, and inspiratory muscle training), also found an increase in both relative FMD (1.05 [95% CI = 0.64, 1.46]) and absolute FMD values (0.98 [95% CI = 0.48, 1.48]). However, they used the standardised mean difference as the ES index, which does not allow us to compare the magnitude of their findings with our own. In contrast, we decided to use a non-standardised ES index because it is easier to interpret from a clinical standpoint. In this regard, there is evidence showing that for every 1% increase of FMD there is a cardiovascular risk reduction of 8–13% [70, 71], highlighting the clinical relevance of exercise-induced endothelial adaptations.

Previous systematic reviews and meta-analyses have demonstrated that aerobic exercise also increases endothelial function in other populations [72–74]. For instance, Ashor et al. [23] reported a FMD increase of 2.79% (95% CI = 2.12, 3.45) in favour of aerobic exercise compared to non-exercise groups in patients suffering from various diseases (e.g. HF, coronary artery disease [CAD], overweight, and peripheral artery disease). Additionally, Manresa-Rocamora et al. [75] reported a FMD improvement of 3.62% (95% CI = 2.62, 4.62) in favour of exercise-based CR in patients with CAD, while Qiu et al. [76] found a FMD increase of 1.77% (95% CI = 0.94, 2.59) in patients with type 2 diabetes. The lower training-induced effect on endothelial function in patients with diabetes could be due to the reduced ability of the endothelium of these patients to produce NO as a consequence of hyperglycaemia [77]. Overall, these findings support the use of aerobic exercise for improving endothelial function and diminishing mortality in healthy individuals and patients suffering from a wide range of pathologies.

Regarding the degree of heterogeneity of the studies we included, our pooled analysis showed high inconsistency of the results, which coincides with the conclusions of previous meta-analyses [20, 23, 75]. Therefore, it was necessary to analyse the influence of potential moderator variables on the improvement of FMD in patients with HFrEF. First, all pooled studies carried out measurements of endothelial function in arteries of the upper limbs (i.e. brachial or radial). Our analyses showed a greater improvement of FMD in studies which assessed endothelial function in the radial artery [45, 51, 54] compared to the brachial artery. This finding is in line with previous evidence. For instance, Agewall et al. [78] reported that FMD was higher in the radial artery than in the brachial artery, regardless of the cuff placement (i.e. forearm and upper arm), which could explain in part the higher training-induced effect found in studies which assessed radial FMD. Second, the effect of aerobic exercise was higher in randomised trials compared to non-randomised studies. Overall, the larger the effect, the lower the likelihood that the results are attributable to confounding factors (e.g. higher activity levels in the experimental group than in the comparison group). Nonetheless, the pooled result of randomised studies was also inconsistent (*I*^2^ = 95.5%). There is evidence that the results of randomised studies with adequate and inadequate allocation concealment could be different [79]. Information regarding the randomisation (i.e. random allocation sequence and allocation concealment) was missing from most of the randomised studies, which could induce selection bias and, therefore, increase the heterogeneity of their findings. Moreover, most of the studies included were judged to be of poor or fair quality, which could lead to bias and partially explain the inconsistency of our findings. This also supports the need for further high-quality trials.

Benda et al. [43] and Kobayashi et al. [50] carried out a cycle ergometer-based CR programme and assessed both upper- and lower-limb endothelial function. They found that FMD improved in the lower limb arteries (i.e. femoral and tibial arteries) but not in the upper limbs, showing an exercise-induced local effect in the trained limbs. It is noteworthy that most of the studies included in our review carried out lower-limb aerobic exercises (e.g. treadmill, cycle ergometer) (see Additional file 1: Table S2). Interestingly, these two studies, which only found improvement in the trained lower limbs, exercised twice a week [43, 50], while the studies that found enhanced upper-limb endothelial function trained three or more days a week. These findings show that a low training frequency (e.g. two sessions a week) is sufficient to induce endothelial adaptations in the trained limbs, but a higher training frequency (e.g. more than two sessions) is necessary to reach systemic vascular adaptations (e.g. increased brachial FMD) beyond the active muscles. These findings have important implications for exercise prescription in CR programmes. In addition, our heterogeneity analyses showed a greater training-induced effect on FMD in studies which carried out a higher number of training sessions per week (e.g. more than three sessions compared to less than four), regardless of the variable scale (i.e. categorical or continuous). These results are in accordance with those previously reported by Ashor et al. [23], who found that the frequency of resistance exercise was positively associated with the improvement in FMD in healthy adults. However, this aspect had not been previously analysed in patient with HF [20, 21].

Other heterogeneity sources should be mentioned. There is evidence about the influence of patients’ characteristics on the training-induced effect on endothelial function [80, 81]. However, aggregated information at the trial level was used to perform meta-analyses, and the relationship between patients’ characteristics and treatment effects was not investigated [82]. Another important source of heterogeneity is the protocol used for FMD acquisition [83]. The recommended post-deflation time to measure FMD is about 180-s [59, 84] because it is assumed that it coincides with the peak arterial dilation. Nevertheless, this time window varied greatly between studies, and some of them used shorter post-dilation imaging (60 to 90-s) [40–42, 44, 47, 50, 57], which could limit the estimation of the true peak dilation.

The effect of aerobic exercise in patients with HFpEF has been poorly investigated, even though endothelial dysfunction is considered an underlying pathophysiological mechanism of HFpEF [85]. Kitzman et al. [49] and Turri-Silva et al. [55], who included patients with HFrEF and HFpEF, analysed the effect of MIT and HIIT, respectively, on endothelial function. The authors found that both aerobic exercise methods did not enhance brachial FMD to a greater degree than non-exercise interventions. These results are in agreement with previous evidence that also showed disappointing effects of drug therapies in patients with HFpEF [86, 87]. However, the low number of studies, as well as the ability of exercise training to improve endothelial dysfunction, warrant future studies exploring the effect of aerobic exercise (i.e. HIIT and MIT) in patients with HFpEF.

Furthermore, since exercise training could be considered a non-pharmacological treatment for improving endothelial function in patients with HFrEF, the efficacy of different “doses” of exercise, in terms of intensity (i.e. HIIT vs. MIT) and exercise modality, should be tested to enable appropriate design of exercise-based CR programmes. In this regard, our subgroup analysis showed that the enhancement of endothelial function occurred after both types of aerobic exercise: MIT (2.95% [95% CI = 1.87, 4.04]) and HIIT (3.53% [95% CI = 0.10, 6.96]) (Additional file 1: Table S4). Moreover, we have found that HIIT improved endothelial function to a greater degree than MIT (2.35% [95% CI = 0.49, 4.22]; Fig. 4) in patients with HFrEF, which is in line with our hypothesis. Ramos et al. [88] and Mattioni Maturana et al. [89] had already reported higher HIIT-induced effects on brachial FMD, but they included healthy people and patients with various diseases (e.g. CAD, diabetes, or obesity). Angadi et al. [39] also found higher FMD improvement after HIIT compared to MIT in patients with HFpEF. Notably, out of all the pooled studies in our meta-analysis, Benda et al. [43] was the only study that did not find any differences between both aerobic exercise methods for enhancing brachial FMD. However, lower-limb exercises were performed twice a week, and a higher training frequency could be necessary to achieve systemic effects. The underlying mechanisms are not fully understood, but it has been speculated that HIIT could induce higher shear stress than MIT on blood vessel walls, promoting greater NO bioavailability [90, 91]. In this regard, there is evidence showing both increased blood flow and shear stress with increasing exercise intensity [92]. In contrast to our finding and previous evidence, Qiu et al. [76] and Pearson and Smart [21] found no differences between both aerobic exercise methods in patients with diabetes and HF, respectively. However, their findings are controversial because some of the studies included performed exercise regimes that did not meet the necessary intensity requirements to consider them as HIIT [18]. Therefore, they did not actually compare HIIT and MIT, rather intermittent versus continuous moderate-intensity training [93]. Thus, to the best of our knowledge, our meta-analysis is the first to provide evidence of the superiority of HIIT for improving endothelial function in patients with HFrEF, and this topic requires future study in patients with HFpEF.

Finally, few studies have investigated the effect of resistance exercise and combined exercise, compared to aerobic exercise, in patients with HF. Munch et al. [52] and Turri-Silva et al. [55] found no differences between resistance exercise and aerobic exercise (i.e. MIT and HIIT, respectively) for enhancing endothelial function. Munch et al. [52] recruited patients with HFrEF, while Turri-Silva et al. [55] included both patients with HFrEF and HFpEF. In contrast, Anagnostakou et al. [38] found an increase in brachial FMD of 4.66% (95% CI = 1.94, 7.38) in favour of combined exercise (i.e. HIIT plus resistance exercise) compared to aerobic exercise (i.e. HIIT) in patients with HFrEF. In line with this finding, although combined exercise and aerobic exercise were not directly compared, Qiu et al. [76] reported in their meta-analysis higher FMD improvement in patients with type 2 diabetes after combined exercise (2.49% [95% CI = 1.17, 3.81]) than after aerobic exercise (1.21% [95% CI = 0.23, 2.19]). On the contrary, Ashor et al. [23] reported that all exercise modalities similarly enhanced endothelial function in patients and healthy individuals. As we can see, there is insufficient evidence in patients with HF to draw solid conclusions about the influence of the exercise modality on the improvement of endothelial function, and the results of previous meta-analyses are controversial. Therefore, future studies should be performed to clarify whether combined exercise is better than aerobic exercise for increasing endothelial function in patients with HF.

### Training-Induced Effect on Vascular Smooth Muscle Function

In accordance with our hypothesis and previous evidence in patients with HF [20] and CAD [75], our findings demonstrate that aerobic-based CR programmes do not enhance vascular smooth muscle function in patients with HFrEF. On the contrary, Liu et al. [94], who included studies performed with patients and healthy individuals, as well as different exercise modalities, reported that exercise training is suitable for improving endothelial-independent dilation. However, heterogeneity was high and exercise-induced adaptations were only found in patients with hypercholesterolemia or rheumatoid arthritis. In addition, Liu et al. [94] found a greater improvement in endothelial-independent dilation in studies which carried out vigorous aerobic exercise. In contrast with these results, Benda et al. [43] and Isaksen et al. [48], who assessed the effect of HIIT compared to non-exercise on vascular smooth muscle function, found no differences. It should be pointed out that none of the previous studies were conducted in patients with HFpEF. Therefore, it seems that aerobic exercise-based CR is not suitable for increasing vascular smooth muscle function in patients with HFrEF. Taking into account that vascular function (i.e. arterial dilation) depends on endothelial and vascular smooth muscle function [13, 95, 96], the lack of improvement of NMD seen in our study confirms that the effect of aerobic exercise on arterial dilation is mediated by an enhancement of endothelial function.

### Strength and Limitations

To the best of our knowledge, the current meta-analysis is the first to study the influence of the aerobic exercise method (i.e. MIT and HIIT) and exercise modality on the improvement of endothelial function in patients with HF. Moreover, we also investigated the influence of other training variables (e.g. training frequency) on the improvement of endothelial function. Exercise characteristics (e.g. intensity of effort bouts) were carefully checked to properly classify studies based on the aerobic exercise method and exercise modality. Finally, robust variance estimation was used to control dependent ES reported from the same research group. Nonetheless, there are some limitations that should be mentioned. Firstly, randomised and non-randomised studies were included, which could increase the inconsistency of our findings. However, heterogeneity analyses were performed based on the study design. Secondly, due to the limited number of studies that investigated the effect of exercise training on endothelial function in patients with HFpEF, we decided not to pool their findings in order to avoid controversial results. Similarly, the low number of studies assessing the effect of resistance exercise and combined exercise did not allow us to conduct pooled analyses to test the influence of exercise modality on endothelial function.

## Conclusions

Our study has evidenced that aerobic-based CR programmes are effective to improve endothelial function in patients with HFrEF, with the potential reduction in mortality and health benefits this implies. Further research is required with HFpEF patients as they are under-represented in the literature. The magnitude of the exercise-induced improvement of endothelial function depends on several training variables which should be considered to design the optimal CR programme. Firstly, a low training frequency (i.e. twice a week) is sufficient to induce vascular adaptations in the trained limbs, but higher frequencies (e.g. three sessions a week) are necessary to produce systemic vascular adaptations. Regarding the aerobic training method, HIIT enhances endothelial function to a greater extent than MIT, although it should be stressed that MIT also improves FMD. Finally, there is not enough evidence to draw solid conclusions about the influence of exercise modality on the enhancement of FMD in patients with HF, which warrants future studies.

From a clinical standpoint, the key question is, do we want patients with HF to train for a limited period or should we aim to promote exercise during their lifetime? If we have a limited period of time (e.g. 8 weeks) and want to achieve the greatest improvement in endothelial function, HIIT is the aerobic exercise method of choice. However, the objective should be to promote exercise for life [97], and a better approach might be to start with MIT and progress to HIIT (principle of training progression). Therefore, we propose that HFrEF patients should begin CR three days a week with MIT (which is sufficient to improve endothelial function). Subsequently, HIIT and resistance exercise should be progressively introduced (e.g. phase 3 CR) to increase the training stimulus over time to avoid plateauing with respect to gains in endothelial function adaptations.

## Supplementary Information

**Additional file 1**. Database search terms. **Table S1**. Items and criteria used for the methodological quality assessment using TESTEX scale. **Table S2**. Intervention and assessment characteristics. **Table S3**. Methodological quality assessment of included studies judged using TESTEX scale. **Table S4**. Analyses of the influence of potential moderator variables on the effect of exercise-based cardiac rehabilitation on relative flow-mediated dilation. **Figure S1**. Forest plot of mean differences between exercise-based cardiac rehabilitation and control group for absolute flow-mediated dilation. **Figure S2**. Forest plot of mean differences between exercise-based cardiac rehabilitation and control group for relative nitroglycerin-mediated dilation.

## Data Availability

The dataset generated from the current study is available from the corresponding author on reasonable request.

## Abbreviations

CG: Control group
CI: Confidence interval
CR: Cardiac rehabilitation
ES: Effect size
FMD: Flow-mediated dilation
HF: Heart failure
HFpEF: Heart failure with preserved ejection fraction
HFrEF: Heart failure with reduced ejection fraction
HIIT: High-intensity interval training
IG: Intervention group
LVEF: Left ventricular ejection fraction
MD: Mean difference
MD_+_: Pooled mean difference
MIT: Moderate-intensity training
NMD: Nitroglycerin-mediated dilation
NO: Nitric oxide
NYHA: New York Heart Association
PRISMA: The Preferred Reporting Items for Systematic Reviews and Meta-Analysis
SD: Standard deviation
TESTEX: The tool for the assessment of study quality and reporting in exercise
VO_2_ peak: Peak oxygen uptake

## References

[1] Savarese G, Lund LH (2017). Global public health burden of heart failure. Card Fail Rev.

[2] Heidenreich PA, Bozkurt B, Aguilar D, Allen LA, Byun JJ, Colvin MM (2022). 2022 AHA/ACC/HFSA guideline for the management of heart failure: executive summary: a report of the American college of cardiology/American heart association joint committee on clinical practice guidelines. Circulation.

[3] McDonagh TA, Metra M, Adamo M, Gardner RS, Baumbach A, Böhm M (2021). 2021 ESC Guidelines for the diagnosis and treatment of acute and chronic heart failure. Eur Heart J.

[4] Del Buono MG, Arena R, Borlaug BA, Carbone S, Canada JM, Kirkman DL (2019). Exercise intolerance in patients with heart failure: JACC state-of-the-art review. J Am Coll Cardiol.

[5] Andrade DC, Arce-Alvarez A, Toledo C, Díaz HS, Lucero C, Quintanilla RA (2018). Revisiting the physiological effects of exercise training on autonomic regulation and chemoreflex control in heart failure: does ejection fraction matter?. Am J Physiol Heart Circ Physiol.

[6] Conraads VM, Van Craenenbroeck EM, De Maeyer C, Van Berendoncks AM, Beckers PJ, Vrints CJ (2013). Unraveling new mechanisms of exercise intolerance in chronic heart failure: role of exercise training. Heart Fail Rev.

[7] Haykowsky MJ, Tomczak CR, Scott JM, Paterson DI, Kitzman DW (1985). Determinants of exercise intolerance in patients with heart failure and reduced or preserved ejection fraction. J Appl Physiol.

[8] Marti CN, Gheorghiade M, Kalogeropoulos AP, Georgiopoulou VV, Quyyumi AA, Butler J (2012). Endothelial dysfunction, arterial stiffness, and heart failure. J Am Coll Cardiol.

[9] Katz SD, Khan T, Zeballos GA, Mathew L, Potharlanka P, Knecht M (1999). Decreased activity of the L-arginine-nitric oxide metabolic pathway in patients with congestive heart failure. Circulation.

[10] Moens AL, Goovaerts I, Claeys MJ, Vrints CJ (2005). Flow-mediated vasodilation: a diagnostic instrument, or an experimental tool?. Chest.

[11] Takishima I, Nakamura T, Hirano M, Kitta Y, Kobayashi T, Fujioka D (2012). Predictive value of serial assessment of endothelial function in chronic heart failure. Int J Cardiol.

[12] Ignarro LJ, Harbison RG, Wood KS, Kadowitz PJ (1986). Activation of purified soluble guanylate cyclase by endothelium-derived relaxing factor from intrapulmonary artery and vein: stimulation by acetylcholine, bradykinin and arachidonic acid. J Pharmacol Exp Ther.

[13] Sandoo A, van Zanten JJ, Metsios GS, Carroll D, Kitas GD (2010). The endothelium and its role in regulating vascular tone. Open Cardiovasc Med J.

[14] Corretti MC, Anderson TJ, Benjamin EJ, Celermajer D, Charbonneau F, Creager MA (2002). Guidelines for the ultrasound assessment of endothelial-dependent flow-mediated vasodilation of the brachial artery: a report of the international brachial artery reactivity task force. J Am Coll Cardiol.

[15] Buckley BJR, Harrison SL, Fazio-Eynullayeva E, Underhill P, Sankaranarayanan R, Wright DJ (2021). Cardiac rehabilitation and all-cause mortality in patients with heart failure: a retrospective cohort study. Eur J Prev Cardiol.

[16] Taylor RS, Long L, Mordi IR, Madsen MT, Davies EJ, Dalal H (2019). Exercise-based rehabilitation for heart failure: cochrane systematic review, meta-analysis, and trial sequential analysis. JACC Heart Fail.

[17] Sarabia JM, Manresa-Rocamora A, Oliveira J, Moya-Ramón M (2018). Influence of the exercise frequency, intensity, time and type according to different training modalities on the cardiac rehabilitation programs. European J Human Mov.

[18] Buchheit M, Laursen PB (2013). High-intensity interval training, solutions to the programming puzzle: part I: cardiopulmonary emphasis. Sports Med.

[19] Fletcher GF, Balady GJ, Amsterdam EA, Chaitman B, Eckel R, Fleg J (2001). Exercise standards for testing and training: a statement for healthcare professionals from the American heart association. Circulation.

[20] Pearson MJ, Smart NA (2017). Effect of exercise training on endothelial function in heart failure patients: a systematic review meta-analysis. Int J Cardiol.

[21] Pearson MJ, Smart NA (2017). Aerobic training intensity for improved endothelial function in heart failure patients: a systematic review and meta-analysis. Cardiol Res Pract.

[22] Hambrecht R, Fiehn E, Weigl C, Gielen S, Hamann C, Kaiser R (1998). Regular physical exercise corrects endothelial dysfunction and improves exercise capacity in patients with chronic heart failure. Circulation.

[23] Ashor AW, Lara J, Siervo M, Celis-Morales C, Oggioni C, Jakovljevic DG (2015). Exercise modalities and endothelial function: a systematic review and dose-response meta-analysis of randomized controlled trials. Sports Med.

[24] Araújo BTS, Leite JC, Fuzari HKB, Pereira de Souza RJ, Remígio MI, Dornelas de Andrade A (2019). Influence of high-intensity interval training versus continuous training on functional capacity in individuals with heart failure: a systematic review and meta-analysis. J Cardiopulm Rehabil Prev.

[25] Li D, Chen P, Zhu J (2021). The effects of interval training and continuous training on cardiopulmonary fitness and exercise tolerance of patients with heart failure-a systematic review and meta-analysis. Int J Environ Res Public Health.

[26] Page MJ, McKenzie JE, Bossuyt PM, Boutron I, Hoffmann TC, Mulrow CD (2021). The PRISMA 2020 statement: an updated guideline for reporting systematic reviews. J Clin Epidemiol.

[27] Smart NA, Waldron M, Ismail H, Giallauria F, Vigorito C, Cornelissen V (2015). Validation of a new tool for the assessment of study quality and reporting in exercise training studies: TESTEX. Int J Evid Based Healthc.

[28] Hedges LV, Olkin I (2014). Statistical methods for meta-analysis.

[29] Higgins JP, Thomas J, Chandler J, Cumpston M, Li T, Page MJ (2019). Cochrane handbook for systematic reviews of interventions.

[30] Borenstein M, Hedges LV, Higgins JP, Rothstein HR (2010). A basic introduction to fixed-effect and random-effects models for meta-analysis. Res Synth Methods.

[31] Higgins JP, Thompson SG, Deeks JJ, Altman DG (2003). Measuring inconsistency in meta-analyses. BMJ.

[32] Cooper H, Hedges LV (1994). The handbook of research synthesis.

[33] Hedges LV, Tipton E, Johnson MC (2010). Robust variance estimation in meta-regression with dependent effect size estimates. Res Synth Methods.

[34] Tipton E (2015). Small sample adjustments for robust variance estimation with meta-regression. Psychol Methods.

[35] Tanner-Smith EE, Tipton E (2014). Robust variance estimation with dependent effect sizes: practical considerations including a software tutorial in Stata and spss. Res Synth Methods.

[36] Peters JL, Sutton AJ, Jones DR, Abrams KR, Rushton L (2008). Contour-enhanced meta-analysis funnel plots help distinguish publication bias from other causes of asymmetry. J Clin Epidemiol.

[37] Sterne JA, Sutton AJ, Ioannidis JP, Terrin N, Jones DR, Lau J (2011). Recommendations for examining and interpreting funnel plot asymmetry in meta-analyses of randomised controlled trials. BMJ.

[38] Anagnostakou V, Chatzimichail K, Dimopoulos S, Karatzanos E, Papazachou O, Tasoulis A (2011). Effects of interval cycle training with or without strength training on vascular reactivity in heart failure patients. J Card Fail.

[39] Angadi SS, Mookadam F, Lee CD, Tucker WJ, Haykowsky MJ, Gaesser GA (2015). High-intensity interval training vs. moderate-intensity continuous exercise training in heart failure with preserved ejection fraction: a pilot study. J Appl Physiol.

[40] Belardinelli R, Capestro F, Misiani A, Scipione P, Georgiou D (2006). Moderate exercise training improves functional capacity, quality of life, and endothelium-dependent vasodilation in chronic heart failure patients with implantable cardioverter defibrillators and cardiac resynchronization therapy. Eur J Cardiovasc Prev Rehabil.

[41] Belardinelli R, Lacalaprice F, Faccenda E, Purcaro A, Perna G (2005). Effects of short-term moderate exercise training on sexual function in male patients with chronic stable heart failure. Int J Cardiol.

[42] Belardinelli R, Lacalaprice F, Ventrella C, Volpe L, Faccenda E (2008). Waltz dancing in patients with chronic heart failure: new form of exercise training. Circ Heart Fail.

[43] Benda NM, Seeger JP, Stevens GG, Hijmans-Kersten BT, van Dijk AP, Bellersen L (2015). Effects of high-intensity interval training versus continuous training on physical fitness, cardiovascular function and quality of life in heart failure patients. PLoS ONE.

[44] Eleuteri E, Mezzani A, Di Stefano A, Vallese D, Gnemmi I, Delle Donne L (2013). Aerobic training and angiogenesis activation in patients with stable chronic heart failure: a preliminary report. Biomarkers.

[45] Erbs S, Höllriegel R, Linke A, Beck EB, Adams V, Gielen S (2010). Exercise training in patients with advanced chronic heart failure (NYHA IIIb) promotes restoration of peripheral vasomotor function, induction of endogenous regeneration, and improvement of left ventricular function. Circ Heart Fail.

[46] Giannattasio C, Achilli F, Grappiolo A, Failla M, Meles E, Gentile G (2001). Radial artery flow-mediated dilatation in heart failure patients: effects of pharmacological and nonpharmacological treatment. Hypertension.

[47] Guazzi M, Reina G, Tumminello G, Guazzi MD (1985). Improvement of alveolar-capillary membrane diffusing capacity with exercise training in chronic heart failure. J Appl Physiol.

[48] Isaksen K, Munk PS, Giske R, Larsen AI (2016). Effects of aerobic interval training on measures of anxiety, depression and quality of life in patients with ischaemic heart failure and an implantable cardioverter defibrillator: a prospective non-randomized trial. J Rehabil Med.

[49] Kitzman DW, Brubaker PH, Herrington DM, Morgan TM, Stewart KP, Hundley WG (2013). Effect of endurance exercise training on endothelial function and arterial stiffness in older patients with heart failure and preserved ejection fraction: a randomized, controlled, single-blind trial. J Am Coll Cardiol.

[50] Kobayashi N, Tsuruya Y, Iwasawa T, Ikeda N, Hashimoto S, Yasu T (2003). Exercise training in patients with chronic heart failure improves endothelial function predominantly in the trained extremities. Circ J.

[51] Linke A, Schoene N, Gielen S, Hofer J, Erbs S, Schuler G (2001). Endothelial dysfunction in patients with chronic heart failure: systemic effects of lower-limb exercise training. J Am Coll Cardiol.

[52] Munch GW, Rosenmeier JB, Petersen M, Rinnov AR, Iepsen UW, Pedersen BK (2018). Comparative effectiveness of low-volume time-efficient resistance training versus endurance training in patients with heart failure. J Cardiopulm Rehabil Prev.

[53] Sales ARK, Azevedo LF, Silva TOC, Rodrigues AG, Oliveira PA, Jordão CP (2020). High-intensity interval training decreases muscle sympathetic nerve activity and improves peripheral vascular function in patients with heart failure with reduced ejection fraction. Circ Heart Fail.

[54] Sandri M, Viehmann M, Adams V, Rabald K, Mangner N, Höllriegel R (2016). Chronic heart failure and aging—effects of exercise training on endothelial function and mechanisms of endothelial regeneration: results from the leipzig exercise intervention in chronic heart failure and Aging (LEICA) study. Eur J Prev Cardiol.

[55] Turri-Silva N, Vale-Lira A, Verboven K, Quaglioti Durigan JL, Hansen D, Cipriano G (2021). High-intensity interval training versus progressive high-intensity circuit resistance training on endothelial function and cardiorespiratory fitness in heart failure: a preliminary randomized controlled trial. PLoS ONE.

[56] Van Craenenbroeck EM, Hoymans VY, Beckers PJ, Possemiers NM, Wuyts K, Paelinck BP (2010). Exercise training improves function of circulating angiogenic cells in patients with chronic heart failure. Basic Res Cardiol.

[57] Wisløff U, Støylen A, Loennechen JP, Bruvold M, Rognmo Ø, Haram PM (2007). Superior cardiovascular effect of aerobic interval training versus moderate continuous training in heart failure patients: a randomized study. Circulation.

[58] Zaky EH, Helmy ZM, Elrefaey BH, Abdusalam IH, Mohamed MAR, Louis O (2018). High intensity interval training versus continuous training on ventricular remodeling in chronic heart failure patients. Biosci Res.

[59] Thijssen DH, Black MA, Pyke KE, Padilla J, Atkinson G, Harris RA (2011). Assessment of flow-mediated dilation in humans: a methodological and physiological guideline. Am J Physiol Heart Circ Physiol.

[60] Dimmeler S, Fleming I, Fisslthaler B, Hermann C, Busse R, Zeiher AM (1999). Activation of nitric oxide synthase in endothelial cells by Akt-dependent phosphorylation. Nature.

[61] Maiorana A, O'Driscoll G, Taylor R, Green D (2003). Exercise and the nitric oxide vasodilator system. Sports Med.

[62] Higashi Y, Sasaki S, Kurisu S, Yoshimizu A, Sasaki N, Matsuura H (1999). Regular aerobic exercise augments endothelium-dependent vascular relaxation in normotensive as well as hypertensive subjects: role of endothelium-derived nitric oxide. Circulation.

[63] Pittner J, Wolgast M, Casellas D, Persson AE (2005). Increased shear stress-released NO and decreased endothelial calcium in rat isolated perfused juxtamedullary nephrons. Kidney Int.

[64] Laufs U, Werner N, Link A, Endres M, Wassmann S, Jürgens K (2004). Physical training increases endothelial progenitor cells, inhibits neointima formation, and enhances angiogenesis. Circulation.

[65] Lenk K, Uhlemann M, Schuler G, Adams V (1985). Role of endothelial progenitor cells in the beneficial effects of physical exercise on atherosclerosis and coronary artery disease. J Appl Physiol.

[66] Koutroumpi M, Dimopoulos S, Psarra K, Kyprianou T, Nanas S (2012). Circulating endothelial and progenitor cells: evidence from acute and long-term exercise effects. World J Cardiol.

[67] Tilling L, Chowienczyk P, Clapp B (2009). Progenitors in motion: mechanisms of mobilization of endothelial progenitor cells. Br J Clin Pharmacol.

[68] Xia WH, Li J, Su C, Yang Z, Chen L, Wu F (2012). Physical exercise attenuates age-associated reduction in endothelium-reparative capacity of endothelial progenitor cells by increasing CXCR4/JAK-2 signaling in healthy men. Aging Cell.

[69] Vuckovic KM, Piano MR, Phillips SA (2013). Effects of exercise interventions on peripheral vascular endothelial vasoreactivity in patients with heart failure with reduced ejection fraction. Heart Lung Circ.

[70] Inaba Y, Chen JA, Bergmann SR (2010). Prediction of future cardiovascular outcomes by flow-mediated vasodilatation of brachial artery: a meta-analysis. Int J Cardiovasc Imaging.

[71] Xu Y, Arora RC, Hiebert BM, Lerner B, Szwajcer A, McDonald K (2014). Non-invasive endothelial function testing and the risk of adverse outcomes: a systematic review and meta-analysis. Eur Heart J Cardiovasc Imaging.

[72] Son Y, Kim K, Jeon S, Kang M, Lee S, Park Y (2017). Effect of exercise intervention on flow-mediated dilation in overweight and obese adults: meta-analysis. Int J Vasc Med.

[73] Pedralli ML, Eibel B, Waclawovsky G, Schaun MI, Nisa-Castro-Neto W, Umpierre D (2018). Effects of exercise training on endothelial function in individuals with hypertension: a systematic review with meta-analysis. J Am Soc Hypertens.

[74] You Q, Yu L, Li G, He H, Lv Y (2021). Effects of different intensities and durations of aerobic exercise on vascular endothelial function in middle-aged and elderly people: a meta-analysis. Front Physiol.

[75] Manresa-Rocamora A, Ribeiro F, Casanova-Lizón A, Flatt AA, Sarabia JM, Moya-Ramón M. Cardiac Rehabilitation Improves Endothelial Function in Coronary Artery Disease Patients. Int J Sports Med. 2022. 10.1055/a-1717-1798 35468652

[76] Qiu S, Cai X, Yin H, Sun Z, Zügel M, Steinacker JM (2018). Exercise training and endothelial function in patients with type 2 diabetes: a meta-analysis. Cardiovasc Diabetol.

[77] Allen JD, Stabler T, Kenjale AA, Ham KL, Robbins JL, Duscha BD (2014). Diabetes status differentiates endothelial function and plasma nitrite response to exercise stress in peripheral arterial disease following supervised training. J Diabetes Complications.

[78] Agewall S, Doughty RN, Bagg W, Whalley GA, Braatvedt G, Sharpe N (2001). Comparison of ultrasound assessment of flow-mediated dilatation in the radial and brachial artery with upper and forearm cuff positions. Clin Physiol.

[79] Odgaard-Jensen J, Vist GE, Timmer A, Kunz R, Akl EA, Schünemann H (2011). Randomisation to protect against selection bias in healthcare trials. Cochrane Database Syst Rev.

[80] Herrington DM, Fan L, Drum M, Riley WA, Pusser BE, Crouse JR (2001). Brachial flow-mediated vasodilator responses in population-based research: methods, reproducibility and effects of age, gender and baseline diameter. J Cardiovasc Risk.

[81] Witte DR, van der Graaf Y, Grobbee DE, Bots ML (2005). Measurement of flow-mediated dilatation of the brachial artery is affected by local elastic vessel wall properties in high-risk patients. Atherosclerosis.

[82] da Costa BR, Juni P (2014). Systematic reviews and meta-analyses of randomized trials: principles and pitfalls. Eur Heart J.

[83] Thijssen DHJ, Bruno RM, van Mil A, Holder SM, Faita F, Greyling A (2019). Expert consensus and evidence-based recommendations for the assessment of flow-mediated dilation in humans. Eur Heart J.

[84] Black MA, Cable NT, Thijssen DH, Green DJ (2008). Importance of measuring the time course of flow-mediated dilatation in humans. Hypertension.

[85] Gevaert AB, Lemmens K, Vrints CJ, Van Craenenbroeck EM (2017). Targeting endothelial function to treat heart failure with preserved ejection fraction: the promise of exercise training. Oxid Med Cell Longev.

[86] Conraads VM, Metra M, Kamp O, De Keulenaer GW, Pieske B, Zamorano J (2012). Effects of the long-term administration of nebivolol on the clinical symptoms, exercise capacity, and left ventricular function of patients with diastolic dysfunction: results of the ELANDD study. Eur J Heart Fail.

[87] Yusuf S, Pfeffer MA, Swedberg K, Granger CB, Held P, McMurray JJ (2003). Effects of candesartan in patients with chronic heart failure and preserved left-ventricular ejection fraction: the CHARM-Preserved Trial. Lancet.

[88] Ramos JS, Dalleck LC, Tjonna AE, Beetham KS, Coombes JS (2015). The impact of high-intensity interval training versus moderate-intensity continuous training on vascular function: a systematic review and meta-analysis. Sports Med.

[89] Mattioni Maturana F, Martus P, Zipfel S, Nieß AM (2021). Effectiveness of HIIE versus MICT in improving cardiometabolic risk factors in health and disease: a meta-analysis. Med Sci Sports Exerc.

[90] Davignon J, Ganz P (2004). Role of endothelial dysfunction in atherosclerosis. Circulation.

[91] Tjønna AE, Lee SJ, Rognmo Ø, Stølen TO, Bye A, Haram PM (2008). Aerobic interval training versus continuous moderate exercise as a treatment for the metabolic syndrome: a pilot study. Circulation.

[92] Thijssen DH, Dawson EA, Black MA, Hopman MT, Cable NT, Green DJ (2009). Brachial artery blood flow responses to different modalities of lower limb exercise. Med Sci Sports Exerc.

[93] Smart NA, Steele M (2012). A comparison of 16 weeks of continuous vs intermittent exercise training in chronic heart failure patients. Congest Heart Fail.

[94] Liu Y, Sun Z, Chen T, Yang C (2021). Does exercise training improve the function of vascular smooth muscle? A systematic review and meta-analysis. Res Sports Med.

[95] Bacakova L, Travnickova M, Filova E, Matějka R, Stepanovska J, Musilkova J (2018). The role of vascular smooth muscle cells in the physiology and pathophysiology of blood vessels. Muscle Cell Tissue Curr Status Res Field.

[96] Vane JR, Anggård EE, Botting RM (1990). Regulatory functions of the vascular endothelium. N Engl J Med.

[97] Sharma S, Pelliccia A, Gati S (2021). The 'ten commandments' for the 2020 ESC guidelines on sports cardiology and exercise in patients with cardiovascular disease. Eur Heart J.

