# Supplementary material for: Effects and Optimal Dose of Exercise on Endothelial Function in Patients with Heart Failure: A Systematic Review and Meta-Analysis
Source: Sports Med Open. 2023 Feb 4;9:8. doi: 10.1186/s40798-023-00553-z (PMC9899305; doi:10.1186/s40798-023-00553-z)
Supplement: Supplementary file 1 — Additional file 1. Database search terms. Table S1. Items and criteria used for the methodological quality assessment using TESTEX scale. Table S2. Intervention and assessment characteristics. Table S3. Methodological quality assessment of included studies judged using TESTEX scale. Table S4. Analyses of the influence of potential moderator variables on the effect of exercise-based cardiac rehabilitation on relative flow-mediated dilation. Figure S1. Forest plot of mean differences between exercise-based cardiac rehabilitation and control group for absolute flow-mediated dilation. Figure S2. Forest plot of mean differences between exercise-based cardiac rehabilitation and control group for relative nitroglycerin-mediated dilation. [file 40798_2023_553_MOESM1_ESM.docx]

**ELECTRONIC SUPPLEMENTARY MATERIAL**

**Database search terms**

(1) Patients. “heart fail*” OR “cardiac fail*” OR “failing heart*” OR “myocardial insufficiency” OR “myocardial fail*” OR “failing myocard*” OR “failure myocard*” OR “heart decompensation” OR “impaired myocardial function” OR “reduced myocardial function” OR HFrEF OR HFpEF

(2) Interventions. “cardiac rehabilitation” OR exercise OR training OR “physical activity” OR “lifestyle change*”

(3) #1 AND #2

(4) Outcomes. “flow-mediated dilat*” OR “nitroglycerin-mediated dilat*” OR “endothelial-dependent dilat*” OR “endothelial-dependent vasodilat*” OR “endothelial-independent dilat*” OR “endothelial-independent vasodilat*” OR “endothelial function” OR FMD OR NMD OR “endothelium-dependent dilat*” OR “endothelium-dependent vasodilat*” OR “endothelium-independent dilat*” OR “endothelium-independent vasodilat*” OR “vascular function” OR “endothelial dysfunction” OR “endothelium dysfunction” OR “brachial artery dilat*” OR “brachial artery vasodilat*”

(5) #3 AND #4

| **Item** | **Question** | **Additional Information** | **Scoring** |
| --- | --- | --- | --- |
| **Study Quality** | | | |
| 1 | Eligibility criteria specified | Eligibility criteria should be specified and fulfilled, and specific diagnostic test values should be provided for all participants | Yes (1) / No (0) |
| 2 | Randomisation specified | A description of the method used to allocate patients into the groups should be provided. Yes, if they are described and truly random (e.g., referring to a random number table, using a computer random number generator, coin tossing, shuffling card or envelopes, throwing dice, drawing of lots, and minimisation) | Yes (1) / No (0) |
| 3 | Allocation concealment | It should be stated if group allocation was concealed. Yes, if group allocation was concealed (e.g., central allocation, sequentially numbered drug containers of identical appearance, and sequentially numbered, opaque, sealed envelopes) | Yes (1) / No (0) |
| 4 | Groups similar at baseline | Baseline data of all participants who were allocated should be presented. There should be no significant or clinical differences between the groups in left ventricular ejection fraction and/or number of comorbidities. Yes, if baseline data are separated by group allocation, presented, and no differences are apparent. | Yes (1) / No (0) |
| 5 | Blinding of assessor for FMD | Yes, if it is clearly stated that assessors of flow-mediated dilation are blinded to the allocation of the patients | Yes (1) / No (0) |

**Table S1.** Items and criteria used for the methodological quality assessment using TESTEX scale

| **Study Reporting** | | | |
| --- | --- | --- | --- |
| 6 | Assessment of outcome measures | The percentage of patients completing the study in the groups should be reported. No, if the number of patients at pre- and post-intervention is not clearly stated or adherence is < 85% (0 points). In contrast, 1 point if this information is clearly stated and adherence > 85%, 1 point if adverse events are reported, and 1 point if exercise attendance is reported | Yes (3) / No (0) |
| 7 | Intention-to-treat analysis | 1 point if all patients completed the intervention (no withdrawal) and 2 points if there are dropouts and intention-to-treat analysis has been used, regardless of the method used to carry out the imputation of the missing values. | Yes (2) / No (0) |
| 8 | Between-group statistical comparisons reported | Between-comparisons should be reported for all included outcomes (i.e., FMD and/or NMD). 1 point if between-group statistical comparisons are reported | Yes (1) / No (0) |
| 9 | Point measures and measures of variability for all reported outcome measures | Point estimates should be provided for all outcomes (i.e., FMD and/or NMD), otherwise this could be deemed selective outcome reporting | Yes (1) / No (0) |
| 10 | Activity monitoring in control groups | 1 point if control patients are asked to report their levels of physical activity and data are presented | Yes (1) / No (0) |
| 11 | Relative exercise intensity remained constant | A periodic assessment of exercise capacity should be conducted to maintain constant the relative intensity. 1 point if intervention length is ≤ 4 weeks (periodic assessment are not necessary) or mid-intervention assessment is performed in studies whose length is > 4 weeks | Yes (1) / No (0) |
| 12 | Exercise volume and energy expenditure reported | 1 point is training variables (i.e., session and programme length, session frequency, intensity, and modality) are clearly reported, regardless of the session (e.g., supervised, unsupervised, and group sessions) | Yes (1) / No (0) |

FMD, flow-mediated dilation; NMD, nitroglycerin-mediated dilation

| **Study (author)** | **Group;**  **EM (AEM)** | **Intervention characteristics** | | **Assessment characteristics** |
| --- | --- | --- | --- | --- |
|  |  | **Setting; length; sessions a week** | **Sessions details (IG) / instructions given to patients (CG)** | **Artery; FMD: cuff placement; occlusion length; occlusion pressure; post-deflation time window**  **NMD: dose; post-administration time window** |
| Anagnostakou et al. (38) | IG;  CE (HIIT) | Centre-based CR; 12 weeks;  3 sessions | RE: 4 exercises; 3 sets × 10 – 12 reps / 30 s (recovery between sets) / quadriceps at 55 – 65% 2 RM (hamstrings – 1 kg), upper limbs at 10 RM  AE: Cycle ergometer; 20 min (session length)^&^ | Brachial (rel and abs);  FMD: distal; 300 s; 220 mmHg; 300 s  NMD: not assessed |
|  | IG;  AE (HIIT) |  | Cycle ergometer; 40 min (session length); 30 s at 50% PPO at steep ramp test (105% PPO at cardiopulmonary exercise test) / 60 s (passive recovery) |  |
| Angadi et al. (39) | IG;  AE (HIIT) | Centre-based CR; 4 weeks;  3 sessions | Treadmill; 10 min at 50% HR peak (WU) + 8 × 2 min at 80 – 85% HR peak / 2 min at 50% HR peak “week 1”; 4 × 4 min at 85 – 95% HR peak / 3 min at 50% HR peak “week 2 – 4” + 5 min at 50% HR peak (CD) | Brachial (rel);  FMD: Based on guidelines (Correti et al., 2002)  NMD: not assessed |
|  | IG^$^;  AE (MIT) |  | Treadmill; 10 min at 50% HR peak (WU) + 15 min at 60% HR peak “week 1”; 30 min at 70% HR peak “week 2 – 4” + 5 min at 50% HR peak (CD) |  |
| Belardinelli et al. (40) | IG;  AE (MIT) | Centre-based CR; 8 weeks;  3 sessions | Cycle ergometer; 15 min of stretching exercise (WU) +  40 min at 60% VO_2_ peak + 5 min loadless (CD) | Brachial (rel);  FMD: distal; 270 s; 240 mmHg; 90 s  NMD: 0.3 mg; 30 s |
|  | CG; NA | NA | No instructions were reported |  |
| Belardinelli et al. (41) | IG;  AE (MIT) | Centre-based CR; 8 weeks;  3 sessions | Cycle ergometer; 15 min of stretching exercise (WU) +  40 min at 60% VO_2_ peak + 5 min loadless (CD) | Brachial (rel);  FMD: distal; 270 s; 240 mmHg; 90 s  NMD: 0.3 mg; 300 s |
|  | CG; NA | NA | No instructions were reported |  |
| Belardinelli et al. (42) | IG;  AE (MIT) | Centre-based CR; 8 weeks;  3 sessions | Cycle ergometer, treadmill, or both; 10 min of calisthenic exercise (WU) +  30 min at 70% VO_2_ peak + 5 min (CD) | Brachial (rel);  FMD: distal; 270 s; 240 mmHg; 90 s  NMD: not assessed |
|  | CG; NA | NA | No instructions were reported |  |
| Benda et al. (43) | IG;  AE (HIIT) | Centre-based CR; 12 weeks;  2 sessions | Cycle ergometer; 10 min at 40% PPO (WU) + 10 × 1 min at 90% PPO (aiming 15 – 17 RPE) / 2.5 min at 30% PPO + 5 min at 30% PPO (CD) | Brachial and femoral (rel);  FMD: distal; 300 s; 220 mmHg; 180 s  NMD: 0.4 mg; NR (only brachial NMD was assessed) |
|  | IG^$^;  AE (MIT) |  | Cycle ergometer; 10 min at 40% PPO (WU) + 30 min at 60 – 75% PPO (aiming 12 – 14 RPE) + 5 min at 30% PPO (CD) |  |
|  | CG; NA | NA | Instructed to continue their normal lifestyle activities |  |

**Table S2.** Intervention and assessment characteristics

**Table S2.** Continued

| **Study (author)** | **Group;**  **EM (AEM)** | **Intervention characteristics** | | **Assessment characteristics** |
| --- | --- | --- | --- | --- |
|  |  | **Setting; length; sessions a week** | **Sessions details (IG) / instructions given to patients (CG)** | **Artery; FMD: cuff placement; occlusion length; occlusion pressure; post-deflation time window**  **NMD: dose; post-administration time window** |
| Eleuteri et al. (44) | IG;  AE (MIT) | Home-based CR; 12 weeks;  5 sessions | Cycle ergometer; 5 min (WU) + 30 min at HR/Watts VT1 + 5 min (CD) | Brachial (rel);  FMD: distal; 300 s; NR; 90 s  NMD: not assessed |
|  | CG; NA |  | Instructed to continue their normal lifestyle activities |  |
| Erbs et al. (45) | IG;  AE (MIT) | Centre- and home-based CR; 12 weeks;  8 sessions | (An inpatient CR programme was performed before starting the study period)  Centre: 1 s/w; 60 min (walking, calisthenics, and ball games)  Home: daily; cycle ergometer; 20 – 30 min at 60% VO_2_ max | Radial (rel);  FMD: proximal; 300 s;  50 mmHg above SBP; 180 s  NMD: not assessed |
|  | CG; NA | NA | Instructed to continue their normal lifestyle activities |  |
| Giannattasio et al. (46) | IG;  AE (MIT) | NR; 8 weeks;  3 sessions | Bicycle; 30 min (intensity NR) | Radial (abs);  FMD: distal; 240 s;  above SBP; 180 s  NMD: 0.3 mg; 300 s |
|  | CG; NA | NA | Instructed to continue their previous standard therapy |  |
| Guazzi et al. (47) | IG;  AE (MIT) | Centre-based CR; 8 weeks;  4 sessions | Cycle ergometer; 5 min (WU) + 30 min at 60 – 80% HR reserve + 5 min (CD) | Brachial (rel);  FMD: distal; 300 s;  50 mmHg above SBP; 90 s  NMD: 0.3 mg; 300 s |
|  | CG; NA | NA | Encouraged to maintain their normal daily activity and attend the clinic every 2 weeks for compliance assessment and avoidance of exercise training |  |
| Isaksen et al. (48) | IG;  AE (HIIT) | Centre-based CR; 12 weeks;  3 sessions | Cycle ergometer or treadmill; 15 min at 60 – 70% HR max (aiming 11 – 13 RPE) (WU) + 4 × 4 min at 85% HR max (aiming 15 – 17 RPE) / 3 min at 60 – 70% HR max + 5 min (CD) + 15 min (strength, stretching, and relaxing) | Brachial (rel and abs);  FMD and NMD: Based on guidelines (Correti et al., 2002) |
|  | CG; NA | NA | No instructions were reported |  |
| Kitzman et al. (49) | IG;  AE (MIT) | Centre-based CR; 16 weeks;  3 sessions | Walking, arm, and cycle ergometer; 10 min (WU) + ≥ 10 min arm ergometer and 40 min (20 min walking + 20 min cycle ergometer) at 70% HR reserve +  10 min (CD) | Brachial (rel and abs);  FMD: distal; 240 s;  50 mmHg above SBP; 180 s  NMD: not assessed |
|  | CG; NA | NA | Received telephone calls every 2 weeks focused on retention, reminders, and not address exercise behaviours |  |
| Kobayashi et al. (50) | IG;  AE (MIT) | Centre-based CR; 12 weeks;  2 – 3 sessions^ | 2 times^; cycle ergometer; 15 min at HR VT1 | Brachial and posterior tibial (rel):  FMD: distal (brachial) and proximal (tibial); 300 s; 200 mmHg; 60 s  NMD: not assessed |
|  | CG; NA | NA | Instructed to continue their normal lifestyle activities |  |

**Table S2.** Continued

| **Study (author)** | **Group;**  **EM (AEM)** | **Intervention characteristics** | | **Assessment characteristics** |
| --- | --- | --- | --- | --- |
|  |  | **Setting; length; sessions a week** | **Sessions details (IG) / instructions given to patients (CG)** | **Artery; FMD: cuff placement; occlusion length; occlusion pressure; post-deflation time window**  **NMD: dose; post-administration time window** |
| Linke et al. (51) | IG;  AE (MIT) | Centre-based CR; 4 weeks;  7 sessions^ | 6 times a day^; cycle ergometer; 10 min at 70% VO_2_peak | Radial (rel and abs);  FMD: proximal; 300 s;  50 mmHg above SBP; 120 s  NMD: intra-arterial infusion (excluded) |
|  | CG; NA | NA | Instructed to continue their sedentary life-style and supervised by their private physician |  |
| Munch et al. (52) | IG;  RE (NA) | Centre-based CR; 6 weeks;  3 sessions | 10 min at 50% PPO (WU) + 4 exercises: 4 sets × 30 s (15 – 20 repetitions) at 25 – 40% 1 RM / 20 s (between sets) and 60 s (between exercises) | Femoral (rel);  FMD: Based on guidelines (Thijssen et al., 2011)  NMD: not assessed |
|  | IG^$^;  AE (MIT) |  | Cycle ergometer; 10 min at 50% PPO (WU) + 35 min at 75% PPO |  |
| Sales et al. (53) | IG;  AE (HIIT) | Centre-based CR; 12 weeks;  3 sessions | Cycle ergometer; progressive work-to-recovery programme (1:1.5 “month 1”, 1:1 “month 2”, 1:0.67 “month 3”) at 5% above HR RCP; EE of 200 kcal/session | Brachial (rel and abs);  FMD: distal; 300 s;  220 mmHg; NR  NMD: not assessed |
|  | IG^$^;  AE (MIT) |  | Cycle ergometer; Intensity between HR VT1 – HR RCP; EE of 200 kcal/session |  |
|  | CG; NA | NA | No instructions were reported |  |
| Sandri et al. (54) | IGs;  AE (MIT) | Centre-based CR; 4 weeks;  6 sessions^ | 4 times every weekday^; cycle ergometer; 5 min (WU) + 15 – 20 min at 70% VO_2_peak + 5 min (CD)  1 group exercise session a week; 60 min (walking, calisthenics, and ball games) | Radial (rel);  FMD: proximal; 300 s;  50 mmHg above SBP; 120 s  NMD: not assessed |
|  | CGs; NA | NA | Patients received usual clinical care by their physicians |  |
| Turri-Silva et al. (55) | IG;  RE (NA) | Centre-based CR; 12 weeks;  3 sessions | 10 min stretching and dynamic movements (WU) + circuit: 3 sets × 6 exercises (upper and lower limbs) at 60% 1 RM “month 1”, 70% 1 RM “month 2”, and 80% 1 RM “month 3”; 6 – 12 repetitions “weeks 1 – 2” and 15 – 20 repetitions “weeks 3 – 4” (within each month) + 5 min (CD) | Brachial (rel);  FMD: proximal; 300 s;  220 mmHg; 180 s  NMD: not assessed |
|  | IG^$^;  AE (HIIT) |  | Cycle ergometer and treadmill (alternately); 10 min at ± 5% HR VT1 + 4 × 3 min at HR VT2– 10% above HR VT2 / 4 min at 10% below HR VT1– HR VT1 +  5 min (CD) |  |
|  | CG;NA | NA | Instructed to keep their routine without changing habits |  |

| **Study (author)** | **Group;**  **EM (AEM)** | **Intervention characteristics** | | **Assessment characteristics** |
| --- | --- | --- | --- | --- |
|  |  | **Setting; length; sessions a week** | **Sessions details (IG) / instructions given to patients (CG)** | **Artery; FMD: cuff placement; occlusion length; occlusion pressure; post-deflation time window**  **NMD: dose; post-administration time window** |
| Van Craenenbroeck et al. (56) | IG;  AE (MIT) | Centre-based CR; 24 weeks;  3 sessions | Cycle ergometer; 60 min at 90% HR RCP | Brachial (rel);  FMD: distal; 240 s;  200 mmHg or at least 50 mmHg above SBP; 270 s  NMD: not assessed |
|  | CG; NA | NA | No instructions were reported |  |
| Wisløff et al. (57) | IG;  AE (HIIT) | Centre- and home-based CR; 12 weeks;  3 sessions | Centre: 2 s/w; uphill treadmill walking; 10 min at 60 – 70% HR peak (WU) + 4 × 4 min at 90 – 95% HR peak / 3 min at 50 – 70% HR peak + 3 min at 50 – 70% HR peak (CD); 38 min (total)  Home: 1 s/w; uphill outdoor walking; 4 × 4 min (intensity that made them to breathe heavily) | Brachial (rel);  FMD: NR (Correti et al., 2002); 300 s; 250 mmHg; 60 s  NMD: 0.5 mg; NR |
|  | IG^$^;  AE (MIT) |  | Centre: 2 s/w; uphill treadmill walking; 47 min at 70 – 75% HR peak  Home: 1 s/w; uphill outdoor walking; 47 min without breathing heavily |  |
|  | CG; NA | NA | Centre: 1 session every 3 weeks; treadmill walking; 47 min at 70% HR peak |  |
| Zaky et al. (58) | IG;  AE (HIIT) | Centre-based CR; 12 weeks;  3 sessions | Cycle ergometer; 5 min at 30% HR peak (WU) + 6 × 1 min at 90 – 95% HR peak / 4 min at 50 – 70% HR peak + 5 min at 30% HR peak (CD) | Brachial (rel);  FMD: proximal; 300 s;  250 mmHg; 120 s  NMD: not assessed |
|  | IG^$^;  AE (MIT) |  | Cycle ergometer; 5 min at 30% HR peak (WU) + 30 min at 60 – 75% HR peak + 5 min at 30% HR peak (CD) |  |

**Table S2.** Continued

abs, absolute values; AE, aerobic exercise; AEM, aerobic exercise method; CD, cool down; CE, combined aerobic and resistance exercise; CG, control group; CR, cardiac rehabilitation; EE, energy expenditure; EM, exercise modality; FMD, flow-mediated dilation; HIIT, high-intensity interval training; HR, heart rate; IG, intervention group; MIT, moderate intensity training; NA, not applicable; NMD, nitroglycerin-mediated dilation; NR, not reported; PPO, peak power output; RCP, respiratory compensation point; RE, resistance exercise; rel, relative values; RM, repetition maximum; RPE, rate of perceived exertion; SBP, systolic blood pressure; s/w, sessions a week; VO_2_, oxygen uptake; VT1, first ventilatory threshold; VT2, second ventilatory threshold; WU, warm-up;

^$^The intervention group was also considered the comparator group

^&^The other details are provided below (aerobic exercise group)

^Several short exercise periods were performed the same day, but it was computed as one exercise session a day

|  | **Study quality** | | | | | **Study reporting** | | | | | | |  |  |
| --- | --- | --- | --- | --- | --- | --- | --- | --- | --- | --- | --- | --- | --- | --- |
| **Study** | **Item 1** | **Item 2** | **Item 3** | **Item 4** | **Item 5** | **Item 6** | **Item 7** | **Item 8** | **Item 9** | **Item 10** | **Item 11** | **Item 12** | **Overall** | **Judgement** |
| Anagnostakou et al. (38) | 1 | 0 | 1 | 1 | 1 | 0 | 0 | 1 | 1 | 0 | 1 | 1 | 8 | Fair |
| Angadi et al. (39) | 1 | 0 | 0 | 1 | 1 | 0 | 0 | 0 | 1 | 0 | 1 | 1 | 6 | Fair |
| Belardinelli et al. (40) | 1 | 0 | 0 | 1 | 1 | 0 | 0 | 1 | 1 | 0 | 0 | 1 | 6 | Fair |
| Belardinelli et al. (41) | 1 | 0 | 0 | 1 | 1 | 3 | 1 | 1 | 1 | 0 | 0 | 1 | 10 | Good |
| Belardinelli et al. (42) | 1 | 1 | 1 | 1 | 1 | 3 | 0 | 1 | 1 | 0 | 0 | 1 | 11 | Good |
| Benda et al. (43) | 1 | 0 | 0 | 1 | 0 | 3 | 0 | 1 | 1 | 0 | 0 | 1 | 8 | Fair |
| Eleuteri et al. (44) | 1 | 0 | 0 | 1 | 1 | 0 | 0 | 0 | 1 | 0 | 0 | 1 | 5 | Poor |
| Erbs et al. (45) | 1 | 1 | 0 | 1 | 1 | 3 | 0 | 1 | 1 | 0 | 0 | 1 | 10 | Good |
| Giannattasio et al. (46) | 0 | 0 | 0 | 1 | 1 | 0 | 0 | 0 | 1 | 0 | 0 | 0 | 3 | Poor |
| Guazzi et al. (47) | 1 | 1 | 1 | 1 | 0 | 0 | 0 | 1 | 1 | 0 | 0 | 1 | 7 | Fair |
| Isaksen et al. (48) | 1 | 0 | 0 | 0 | 0 | 3 | 0 | 1 | 1 | 0 | 0 | 1 | 7 | Fair |
| Kitzman et al. (49) | 1 | 0 | 0 | 1 | 1 | 0 | 0 | 1 | 1 | 0 | 0 | 1 | 6 | Fair |
| Kobayashi et al. (50) | 1 | 0 | 0 | 1 | 1 | 2 | 1 | 1 | 1 | 0 | 0 | 1 | 9 | Good |
| Linke et al. (51) | 1 | 0 | 0 | 1 | 0 | 0 | 0 | 1 | 1 | 0 | 1 | 1 | 6 | Fair |
| Munch et al. (52) | 1 | 1 | 1 | 1 | 0 | 3 | 0 | 1 | 1 | 0 | 0 | 1 | 10 | Good |
| Sales et al. (53) | 1 | 0 | 0 | 1 | 0 | 0 | 0 | 1 | 1 | 0 | 0 | 1 | 5 | Poor |
| Sandri et al. (54) | 1 | 1 | 1 | 1 | 1 | 2 | 1 | 1 | 1 | 0 | 1 | 1 | 12 | Excellent |
| Turri-Silva et al. (55) | 1 | 1 | 1 | 1 | 0 | 0 | 0 | 1 | 1 | 0 | 0 | 1 | 7 | Fair |
| Van Craenenbroeck et al. (56) | 1 | 0 | 0 | 1 | 1 | 0 | 0 | 1 | 1 | 0 | 0 | 1 | 6 | Fair |
| Wisløff et al. (57) | 1 | 1 | 0 | 1 | 0 | 3 | 0 | 1 | 0 | 0 | 0 | 1 | 8 | Fair |
| Zaky et al. (58) | 1 | 0 | 0 | 1 | 0 | 0 | 0 | 1 | 1 | 0 | 0 | 1 | 5 | Poor |

**Table S3.** Methodological quality assessment of included studies judged using TESTEX scale

Item 1, eligibility criteria specified; Item 2, randomisation specified; Item 3, allocation concealment; Item 4, group similar at baseline; Item 5, blinding of assessor for flow-mediated dilation; Item 6, outcome measures assessed in 85% of patients; Item 7, intention-to-treat analysis; Item 8, between-group statistical comparisons reported; Item 9, point measures and measures of variability for all reported outcome measures; Item 10, activity monitoring in control groups; Item 11, relative exercise intensity remained constant; Item 12, exercise volume and energy expenditure

| - **Categorical variables** | | | | | **Test for subgroup differences** | |
| --- | --- | --- | --- | --- | --- | --- |
| Moderator | **Category** | | **K** | **MD (95% CI)** | ***Chi^2^*** | ***p^a^*** |
| Study design | Randomised | | 14 | 3.59 (2.43, 4.76) | 4.21 | .040 |
|  | Non-randomised | | 4 | 1.21 (–0.74, 3.17) |  |  |
| Artery | Brachial | | 14 | 2.51 (1.36, 3.65) | 7.07 | .008 |
|  | Radial | | 4 | 5.25 (3.58, 6.92) |  |  |
| Sex | Males | | 6 | 3.76 (2.02, 5.49) | 0.79 | .375 |
|  | Mixed sample | | 12 | 2.75 (1.36, 4.14) |  |  |
| Aerobic exercise method | HIIT | | 4 | 3.53 (0.10, 6.96) | 0.10 | .754 |
|  | MIT | | 14 | 2.95 (1.87, 4.04) |  |  |
| Sessions a week | > 3 sessions | | 6 | 4.41 (2.88, 5.94) | 3.59 | .050 |
|  | ≤ 3 sessions | | 12 | 2.46 (1.15, 3.78) |  |  |
| - **Continuous variables** | | | | |  | |
| **Moderator** | | **K** | ***B* (95% CI)** | | ***Z*** | ***p*** |
| Intervention length, weeks | | 18 | –0.11 (–0.36, 0.13) | | –0.91 | .364 |
| Sessions a week | | 18 | 0.82 (0.29, 1.35) | | 3.04 | .002 |
| Total number of sessions (×10) | | 18 | 0.04 (–0.02, 0.09) | | 1.41 | .159 |

**Table S4.** Analyses of the influence of potential moderator variables on the effect of exercise-based cardiac rehabilitation on relative flow-mediated dilation

*B, B* regression coefficient; *Chi^2^*, chi-square statistic; CI*,* confidence interval; HIIT, high-intensity interval training; *I^2^*, heterogeneity index; K*,* number of analysis units; MD, mean difference; MIT, moderate intensity training; *p,* probability level associated to the absolute value of *Z* statistic for *B* regression coefficient; *p^a^*, probability level associated to *chi-squared* statistic; *Z, Z* statistic for *B* regression coefficient


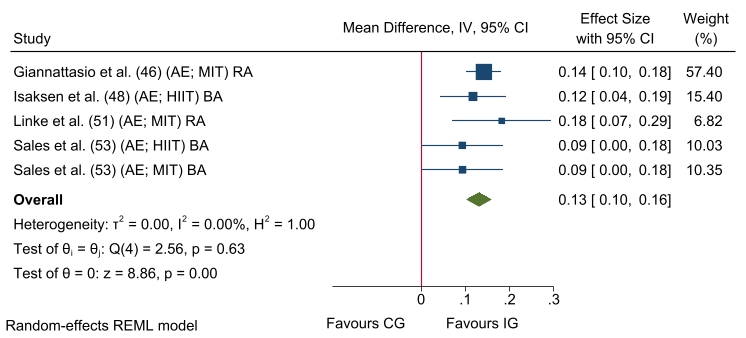


**Figure S1.** Forest plot of mean differences between exercise-based cardiac rehabilitation and control group for absolute flow-mediated dilation. AE, aerobic exercise; BA, brachial artery; CG, control group; CI, confidence interval; HIIT, high-intensity interval training; IG, intervention group; IV, inverse variance; MIT, moderate intensity training; RA, radial artery


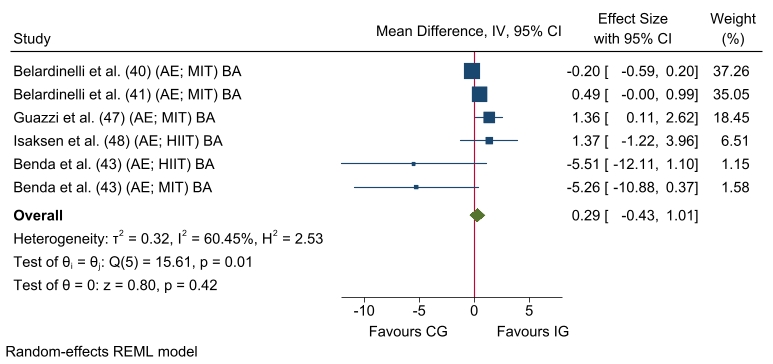


**Figure S2.** Forest plot of mean differences between exercise-based cardiac rehabilitation and control group for relative nitroglycerin-mediated dilation. AE, aerobic exercise; BA, brachial artery; CG, control group; CI, confidence interval; HIIT, high-intensity interval training; IG, intervention group; IV, inverse variance; MIT, moderate intensity training
